# The *Trichoderma atroviride* putative transcription factor Blu7 controls light responsiveness and tolerance

**DOI:** 10.1186/s12864-016-2639-9

**Published:** 2016-05-04

**Authors:** José E. Cetz-Chel, Edgar Balcázar-López, Edgardo U. Esquivel-Naranjo, Alfredo Herrera-Estrella

**Affiliations:** Laboratorio Nacional de Genómica para la Biodiversidad, CINVESTAV Sede Irapuato, Km 9.6 Libramiento Norte Carretera Irapuato-León, 36821 Irapuato, Guanajuato Mexico; Present Address: Unit for Basic and Applied Microbiology, Faculty of Natural Sciences, Autonomous University of Querétaro, Querétaro, 76230 Mexico

**Keywords:** BLR, RNA-seq, Photoconidiation, Nitrogen metabolism, Glucose, Growth, Light

## Abstract

**Background:**

Most living organisms use sunlight as a source of energy and/or information about their environment. Consequently, they have developed mechanisms to sense light quality and quantity. In the fungus *Trichoderma atroviride* blue-light is perceived through the Blue Light Regulator Complex, which in turn up-regulates a set of genes (*blu*) and down-regulates another set (*bld*), triggering asexual reproduction. To gain insight into this process, we characterized the *blu7* gene, which encodes a protein containing a C2H2 zinc finger domain.

**Results:**

Δ*blu7* mutants show reduced conidiation at low light fluences, which is still clear even when exposed to saturating light. For the first time we show a genome wide survey of light regulated gene expression in *T. atroviride*, including RNA-seq analyses of the wild type and the Δ*blu7* strains after brief exposure to blue-light. Our data show a reduction in the number of induced genes and an increase in down-regulated genes in the mutant. Light activates stress responses and several metabolic processes in the wild type strain that are no longer activated in the mutant. In agreement with the misregulation of metabolic processes, continuous exposure to white light strongly inhibited growth of the ∆*blu7* mutant, in a carbon source dependent fashion. RNA-seq analyses under constant white light using glucose as sole carbon source revealed that localization and transport process present the opposite regulation pattern in the ∆*blu7* and wild type strains. Genes related to amino acid, sugar and general transporters were enriched in the induced genes in the mutant and the repressed genes of the wild type. Peptone supplemented in the media restored growth of the ∆*blu7* mutant in constant light, suggesting a role of Blu7 in the regulation of nitrogen metabolism in the presence of light.

**Conclusions:**

Blu7 appears to regulate light sensitivity in terms of induction of conidiation, and to play a major role in supporting growth under continuous exposure to light. The diminished conidiation observed in ∆*blu7* mutants is likely due to misregulation of the cAMP signaling pathway and ROS production, whereas their low tolerance to continuous exposure to light indicates that Blu7 is required for adaptation.

**Electronic supplementary material:**

The online version of this article (doi:10.1186/s12864-016-2639-9) contains supplementary material, which is available to authorized users.

## Background

Most organisms can perceive light as a signal and the response to this stimulus depends on the length of exposure and light quality, serving as a cue of environmental conditions [1]. Fungi can perceive a wide range of light wavelengths, from far red to ultraviolet (UV) light [2–4]. Many reports on light responses in fungi are related to reproduction, which determines whether they should enter asexual or sexual reproduction or neither [4–8].

The best-characterized photoreceptor in fungi is the White Collar Complex (WCC) of *Neurospora crassa*. This blue light photoreceptor WCC, formed by the White Collar (WC-1, WC-2) proteins, regulates pigmentation, circadian rhythm, conidiation and phototropism of perithecial beaks [9–11]. The WCC of *N. crassa* controls all light responses characterized so far in this fungus, despite the presence of red (phytochromes), UV (cryptochromes) or green (opsin) photoreceptors in its genome [12–15]. However, a cryptochrome dependent oscillator (CDO) driving rhythmic spore development under constant light, where the Frequency/WCC oscillator does not operate, was recently described [16]. The *cog-1* (*cry-dependent oscillator gate-1)* mutation uncovered this CRY (Cryptochrome) dependent oscillator in *N. crassa*, which regulates light responsive genes independently of the WCC. Similarly, as no obvious biological functions for Phy-1/Phy-2 or Nop-1 are reported, their role might be masked by the WCC function. Moreover, in *Aspergillus nidulans* the phytochrome (FphA) is involved in repression of sexual development and mycotoxin production by red light, whereas the LreA and LreB proteins (orthologues of the WC proteins) stimulate both [6, 17, 18].

Asexual reproduction induced by light in *T. atroviride* results in the formation of the so called “conidiation ring” in the periphery of the colony. The BLRC, formed by the BLR1 and BLR2 proteins, is responsible for the perception of blue light [19]. Like their *N. crassa* counterparts, the BLR proteins have GATA zinc finger DNA binding domains, that allows them to act as transcription factors, and PAS domains to form a protein complex. The BLRC controls the transcriptional response to light and photoconidiation [19–21]. In *T. atroviride* a set of genes targeted by the BLRC was identified by microarray analysis after a 30 min pulse of white light [22]. Rosales et al. [22] reported only 40 differentially expressed genes, 30 blue light up-regulated (*blu*) and 10 blue light down-regulated (*bld*). From the 40 light regulated genes, only the *blu7* gene was predicted as a putative transcription factor (TF) encoding a C2H2 zinc finger DNA binding domain protein. In this sense, microarray analyses covering the complete genome of the fungus *N. crassa* revealed the activation of six genes encoding transcription factors in response to light [15]. However, recently, high-throughput RNA sequencing showed that there are 58 light responsive transcription factors in *N. crassa* [23]. Furthermore, transcriptional regulation by the *N. crassa*’s WCC in response to a brief pulse (8 min) of white light was recently analyzed by Chip-Seq, uncovering more than 400 genes regulated by this complex, including 24 transcription factors (TFs) as direct targets [24]. Currently, only six of the transcription factors, (2 GATA (WC-1, SUB-1), 2 C2H2 (CSP-1, SAH-1) and 2 Zn_2_Cys_6_ (VAD-3 & Cutinase TF-1ß)) reported by Chen et al., [15] have been studied in response to constant white light exposure. These transcription factors regulate genes after illumination at early or late stages [15]. However, only the absence of the GATA factor *sub-1* (*submerged protoperithecia-1*) showed the expected lack of expression in late light regulated genes after illumination with white light, as a component of a second transcriptional cascade [15]. Despite the lack of late light regulated genes in ∆*sub-1*, asexual reproduction and carotenoid accumulation after illumination is similar to that of the wild type (WT); suggesting a main role of *sub-1* in sexual reproduction, acting as a repressor of protoperithecia formation [25]. The recent findings by RNA-seq analysis of the light response in *N. crassa* revealed a complex regulation of gene expression during illumination, unveiling down-regulated genes previously not found by microarrary analysis. In addition, TFs negatively regulated after the light treatment were discovered, integrating more pieces to the puzzle of the light response in this fungus [23].

In *A. nidulans* the LreA and LreB proteins regulate 425 genes positively and 108 genes negatively in response to a brief pulse of light, representing 5 % of the genome [26]. Expression of the *flbC* gene, a C2H2 TF involved in conidiation, depends on the complex photoreceptor system integrated by the Light Response proteins LreA and LreB as well as the red light photoreceptor FphA. *flbC* activation turns on the expression of the transcription factor *brlA*, a well-known C2H2 TF regulator of conidiation [27]. The photoreceptor complex also activates the expression of *flbB* and *flbD* encoding bZIP and cMYC TFs, which also regulate the expression of *brlA*, to promote the morphological transition of vegetative growth to conidiophores in *A. nidulans* [26]. BrlA regulates the transition of the elongated hyphae to metulae, which in turn activates *abaA* expression [28]. AbaA controls the correct formation of conidial beaks, whose maturation is reached upon activation of WetA [29]. *T. atroviride* has one orthologue of the *flbC* gene as putative transcriptional activator but its expression does not appear to be affected by light, similarly to what is observed in *A. fumigatus* [30]. Despite the fact that there is no obvious *brlA* orthologue in the genome of *T. atroviride*, homologues of AbaA (37 % identity with *A. nidulans*) and WetA (*A. nidulans*, 66 % identity of the C-terminal region) have been identified. Overall, it is clear that there are undiscovered light transcriptional response pathways in *T. atroviride*. At least part of such pathways must be BLRC targets, such as the putative C2H2 transcription factor Blu7, acting downstream in response to light. The rapid activation of *blu7* expression, by the Blr proteins, showed a maximum at 15 min both under constant illumination and after a pulse of blue light, suggesting a role in the control of early light regulated genes [31].

The cAMP signaling pathway is involved in several processes in fungi, such as growth, reproduction and nutrient utilization [32, 33]. In *T. atroviride* light stimulates cAMP synthesis and asexual reproduction is stimulated by addition of extracellular cAMP both in the dark and in light on rich medium, but requires the presence of the Blr1 and Blr2 proteins. Intriguingly, the induction of asexual sporulation by sudden carbon starvation also requires the presence of the Blr1 and Blr2 proteins, but the addition of extracellular cAMP triggers conidiation even in the absence of the *blr1* or *blr2* genes [34]. In addition, extracellular cAMP changes the degree of stimulation of conidiation provoked by different carbon sources in the *blr* mutants of *T. atroviride*, both in a positive and negative way [35]. Furthermore, the carbon source available and the Blr1 or Blr2 proteins act together to stimulate growth and conidiation in the presence of light or in the dark [35, 36]. These data led to the proposal that the cAMP-signaling pathway regulates conidiation genes through the action of the cAMP dependent kinase (PKA) in coordination with the BLRC in response to light [34]. On top of that, the BLRC dependent PAS domain protein Envoy of *Trichoderma reesei*, another photoreceptor, regulates cAMP production in the presence of light mainly by inhibition of the corresponding phosphodiesterase, linking regulation of asexual reproduction and nutrient signaling by modulating the expression of the G-proteins Gna1 and Gna3 [37].

The present work describes the role of the putative C2H2 zinc finger transcription factor Blu7 in the response to light. RNA-seq analysis of the *blu7* gene replacement mutants was carried out after a pulse of blue light (100 μmolm^−2^) to evaluate its role in photoconidiation. The rapid accumulation of the *blu7* mRNA in response to light led us to hypothesize that it could be part of a transcriptional cascade resulting in asexual reproduction. The Blr1 and Blr2 dependent induction of asexual reproduction by glucose starvation led us ask if the Blu7 C2H2 zinc finger protein is involved in this process. Hence, we evaluated the transcriptional response of the ∆*blu7* mutant when exposed to constant white light on glucose as a carbon source. Surprisingly, the transcriptional analysis of the light response uncovered a role of the *blu7* gene in nitrogen regulation in a glucose dependent way.

## Results

### Blu7 is commonly found among Hypocreales

Blue light perception in *T. atroviride* through the Blr1 and Blr2 proteins activates transcription factors to control subsequent events of a transcriptional cascade. One of this putative BLR dependent transcription factors is encoded by the up-regulated gene *blu7* [22]. The previously reported *blu7* cDNA (642 bp) [22], differed from the gene prediction based on genome sequencing (Id 138208; http://genome.jgi.doe.gov/cgi-bin/dispGeneModel?db=Triat2&id=138208), in that it contained a shorter open reading frame (213 aa). To establish what was the actual gene, we sequenced the cDNA, which corresponded to a 3002 bases long mRNA (GenBank Id KU666056). The CDS corresponded with that of another predicted gene (Id 284873; http://genome.jgi.doe.gov/cgi-bin/dispGeneModel?db=Triat2&id=284873; Additional file 1A), and encodes a 537 amino acid protein with a C2H2 zinc finger DNA binding domain at the C-terminal region, which also contains a Nuclear Localization signal (NLS), two proline rich (ProRich) motifs at the N-terminal region, and a glutamine rich region predicted by Motif-Scan (http://myhits.isb-sib.ch/cgi-bin/motif_scan) as a putative activation domain [22].

To get insight into the putative function of the Blu7 protein we searched for homologues by BLAST against the non-redundant database (nr) of the NCBI. Orthologues of the Blu7 protein are present in many, but not all ascomycetes, and they are more commonly found in the Hypocreales, being *Trichoderma spp.*, *Fusarium, Metarhizium* and *Colletotrichum spp.* the most representative. Interestingly, we did not find orthologues of Blu7 in the *N. crassa* or *A. nidulans* genomes*.*

### Absence of the *blu7* gene results in reduced photoconidiation in *T. atroviride*

We replaced part of the coding sequence of *blu7*, covering the polyQ, ProRich, and zinc finger C2H2 type domain at the C-terminal region of the originally predicted 213 amino acid protein [22], by a cassette containing the selectable marker *hph* using the PCR double-joint protocol [38]. Replacement of this locus was validated by PCRs flanking the replacement cassette and confirmed by Southern blot in six independent transformants (Additional file 2A). Although deletion of the *blu7* locus was not complete, given that the replacement eliminated the most relevant motifs, we expected to generate a loss of function allele for this putative transcription factor (Additional file 2B).

The growth rate of the mutants was not affected in the dark, neither under constant white light illumination (3.2 μmolm^−2^s^−1^), as compared to the WT (Additional file 3A). However, a clear growth delay of the three independent ∆*blu7* mutants tested was observed when the strains were grown under constant blue light (4.9 μmolm^−2^s^−1^, Additional file 3B). Noticeably, during the first 36 h continuous illumination provoked the strongest effect on growth but by the end of the assay all colonies had the same diameter, suggesting that the mutants do not adapt to or tolerate light normally, and might, upon prolonged exposure to light, be able to compensate for this defect. In contrast, when grown under photoperiods of 12 h blue light-darkness (4.9 μmolm^−2^s^−1^) during 72 h, the ∆*blu7* mutants showed the same growth as the WT (Additional file 3C). We also observed that 90 % more conidia were produced under constant blue (4.9 μmolm^−2^s^−1^) compared to white light (3.2 μmolm^−2^s^−1^) in the WT, whereas in the ∆*blu7* mutants the level of conidiation in blue or white light was similar (Additional file 3D). Production of conidia by the ∆*blu7* mutants under constant white light was 50 % of that observed for the WT, whereas when exposed to blue light conidia production of the mutant dropped to 33 % of that of the WT (Additional file 3D).

**Fig. 1:**
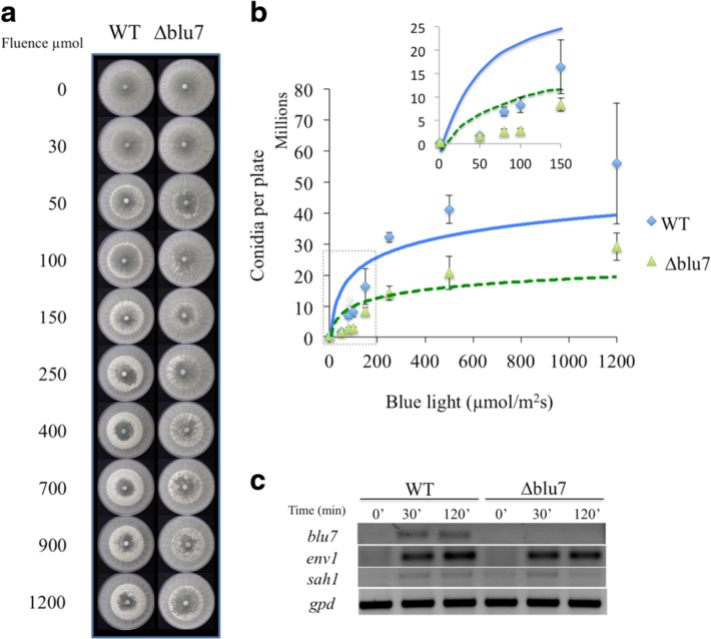
Photoconidiation of the WT and ∆*blu7* strains after a pulse of blue light. **a** Induction of conidiation ring in WT and ∆*blu7* mutant strains in response to increasing blue light fluence, as indicated. **b** Conidia production of the WT (squares) and the ∆*blu7* (triangles) mutant at different blue light fluence. The inset is a zoom view of the induction of conidiation at fluences lower than 200 μmolm^−2^. The strains were cultivated during 36 h in the dark, then exposed to a pulse of blue light of 0, 50, 80, 100, 250, 500, 1200, 2400, 4800, 9600 and 11000 μmolm^−2^. **c** Semiquantitative RT-PCR of the *blu7, env1* and *sah1* genes after a pulse of blue light in the WT and the ∆*blu7* mutant; *gpd* was used as a control. Replicates of three independent assays were used to evaluate conidia production by the mutant and WT strains. A *t*-test was applied to data with significant differences set at *P* < 0.05. A logarithmic base 10 tendency line was obtained for the data of the WT (blue line) and ∆*blu7* mutant (green line)

Since the BLRC is required to induce the formation of a conidiation ring after a pulse of blue light, and *blu7* expression is BLR dependent, we evaluated the production of asexual spores of the mutants when exposed to varying blue light fluence. The conidiation ring was not formed at light fluences lower than 150 μmolm^−2^ in the ∆*blu7* mutants. Nevertheless, at higher blue light fluences the conidiation ring is observed, but the mutants never reach the yield of conidia of the WT (Fig. 1a, b). Production of conidia is already detectable upon exposure to 50 μmolm^−2^ of blue light in the WT, whereas the ∆*blu7* mutants require 150 μmolm^−2^ to trigger the conidiation process, pointing to either a light perception or downstream signaling defect (Fig. 1a, b). However, the two strains appear to require approximately the same amount of photons to reach half-saturation of the response, suggesting that there is no such a defect in light perception. We then analyzed the expression of the *blu7, env1* and, *short aerial hyphae 1* (*sah1*) genes after a pulse of 100 μmolm^−2^ of blue light by semiquantitative PCR (Fig. 1c). mRNA of *blu7* was observed even after 120 min of light induction in the WT and, as expected, it was not detected in the ∆*blu7* mutant. Expression of the *sah1* and *env1* genes had the same profile in the WT and the ∆*blu7* strain after light induction, suggesting that light perception by the BLRC is functional. Thus, the absence of conidiation after exposure to 100 μmolm^−2^ of blue light is possibly due to the control of a particular set of genes by Blu7.

### The transcriptional response to light of the ∆*blu7* mutant

**Fig. 2:**
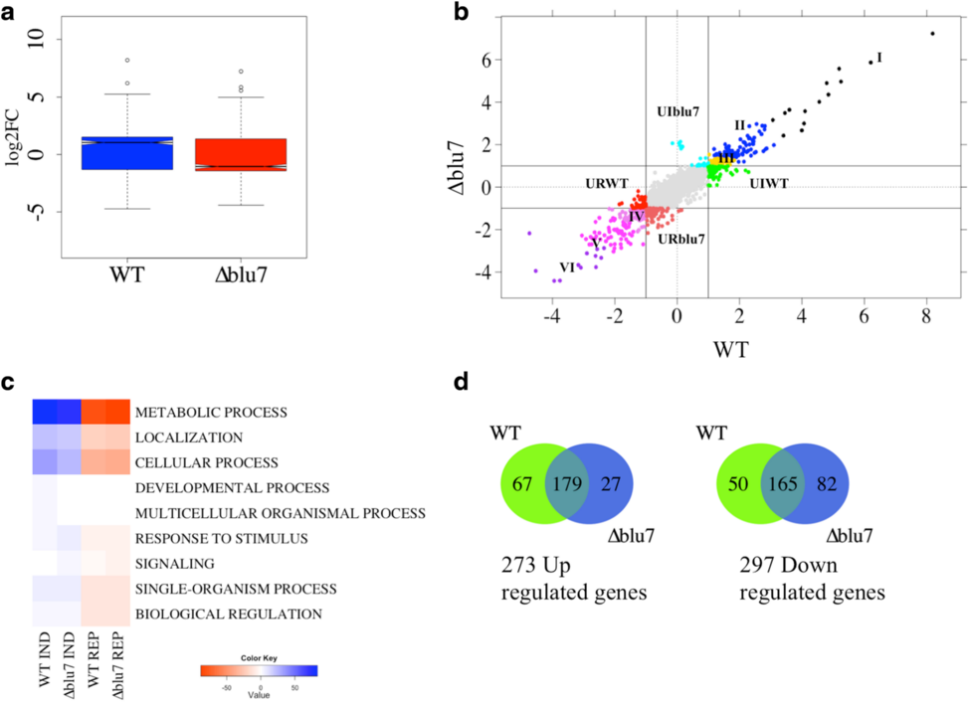
Transcriptional response of the WT and ∆*blu7* strains 30 min after a pulse of blue light. **a** Boxplot of log2FC of the total differential light regulated genes in the WT and ∆*blu7* strains, compared to dark control of each strain. **b** Plot of the WT vs ∆*blu7* by log2FC, the resulting 6 clusters of the common regulated genes are indicated with roman numbers. The light induced genes present only in the mutant are localized at the positive Y-axis depicted in cyan and the repressed genes in dark orange in the negative Y-axis. The light induced genes present only in the WT are observed in green at the positive X-axis and the repressed genes are in red at the negative X-axis. **c** BLAST2GO annotation by biological process of the light regulated genes in the WT or ∆*blu7* mutant strain. **d** Venn diagram showing the overlap in up-regulated (left) and down-regulated (right) genes that respond in the WT and ∆*blu7* mutant strain

Given that the ∆*blu7* mutants are unable to form the conidiation ring at 100 μmolm^−2^ blue light, we decided to analyze the transcriptomes of the WT and a ∆*blu7* mutant under this condition to identify genes whose expression could be affected in the mutant, and that could be involved in asexual reproduction. A total of 453 genes were differentially expressed in the ∆*blu7* and 461 in the WT in light as compared to controls kept in the dark [cutoff **F**old **C**hange (FC) >2 (log2 |FC| >1), False Discovery Rate (FDR) < 0.01; Additional file 4]. The WT strain showed more up-regulated genes (246) than the ∆*blu7* mutant (206), while the ∆*blu7* mutant presented more repressed genes (247) than the WT (215). The expression levels of the main light responsive genes in the WT and ∆*blu7* showed similar degree of induction or repression. However, in the WT the light regulated genes in average are more strongly induced (Fig. 2a).

Hierarchical clustering of the differentially expressed genes, based on expression level, results in six main clusters for the total light responsive genes, as compared to their corresponding dark control (Additional file 5A; Fig. 2b). The most responsive genes, up- or down-regulated, were grouped in Clusters I and VI (Additional file 4 and Additional file 5A). Cluster II contained induced genes ranging from FC 2.3 to 8. Cluster III contained genes up-regulated in *∆blu7* in a FC range between 2 and 2.5, whereas in the WT ranged from FC 2 to 3.5. In Cluster IV we found the less repressed genes. In Cluster V the level of repression was similar in both strains, although not in the same genes. Homologues of previously reported light responsive genes in fungi were found distributed in all clusters.

Among the genes regulated in common in both strains we found six genes more strongly induced in ∆*blu7* (Additional file 6) and 26 genes with higher induction in the WT (Additional file 6). On the other hand, among this set of genes we found 32 more strongly repressed in ∆*blu7* (Additional file 7) and 16 genes with stronger repression in the WT (Additional file 7).

In Cluster I we found two alleles (Id. 300570 and Id.: 529689) orthologous to the *N. crassa ccg-1/grg1* gene (***c****lock****c****ontrolled****g****ene-****1***), one of them (Id. 300570) showed the highest level of expression in both the WT (FC = 292.3) and the ∆*blu7* (FC = 149.4) mutant (Additional file 4). Interestingly *env1*, a gene related to photoadaptation of light regulated genes, was more strongly induced in the WT (FC = 73.9) than in the ∆*blu7* (FC = 58) mutant (Z-score < 0.2). The remaining 7 genes in Cluster I were related to catalytic activity, protein binding and nucleotide binding with similar expression profile in the WT and the ∆*blu7* strain. In Cluster II we found an orthologue of the *frq* gene of *N. crassa* (Id. 131340), involved in circadian clock regulation, and the *blu17* gene (Id. 160158), an orthologue of the *N. crassa al-3* gene (encoding a geranylgeranyl pyrophosphate synthase). The *frq* gene had the same level of expression in both strains; but the orthologue of *al-3* was slightly more strongly induced in the WT (WT FC = 4.2; ∆*blu7* FC = 2.9). Looking into the transcription factor encoding genes, we detected four in this cluster, three of them corresponding to Zn_2_Cys_6_ zinc finger proteins not yet studied, and the *cp2* gene (Id. 319089), an orthologue of the *grh-like* gene of *N. crassa* involved in the release of spores [39]. Only *cp2* showed a slightly stronger induction in the ∆*blu7* (FC = 3.8) than in the WT (FC = 3.1). Interestingly, the DNA photolyase *phr1* (Id. 86846) and a putative base excision DNA repair (Id. 26345) protein encoding genes had stronger induction in the WT than in the mutant, both genes related to DNA damage response (Additional file 4).

In Cluster III we detected two transcription factors, the C2H2 zinc finger protein encoded by *azf1* (Id. 165197) and the GATA type *sub1* gene (Id. 258818). Induction of the *azf1* gene, involved in glucose growth regulation by cyclin control in *Saccharomyces cerevisiae* [40, 41] was stronger in the WT (FC = 3) than in ∆*blu7* (FC = 2.1). Induction of *sub1* was higher in the ∆*blu7* mutant (FC = 2.86) than in the WT (FC = 2).

In Cluster IV we found nine genes that were more repressed in the WT than in the mutant, and 13 genes more repressed in the mutant than in the WT (Additional file 4). The genes encoding a calcipresin family protein (Id. 299076), a Ste20 like protein kinase (Id. 242521) were among the most repressed ones in the WT. Calcipresin is a negative regulator of the Calcineurin-Calmodulin phosphatase in yeast and mammals [42, 43]. In *Aspergillus fumigatus* and *Botrytis cinerea*, calcipresin participates as a positive regulator of hyphal growth [44, 45]. In Cluster V, 6 genes were more repressed in the WT than in the mutant, and 17 genes more repressed in ∆*blu7* than in the WT. The genes with stronger repression in both strains were detected in Cluster VI (violet, Fig. 2b). The remaining genes were repressed to a similar extent in the *∆blu7* and the WT (Additional file 4).

Most of the induced genes in the WT and ∆*blu7* mutant belong to the GO-term metabolic process (Fig. 2c). We observed the same subcategories of the metabolic process in the WT and ∆*blu7* strains. However, genes belonging to the lipid metabolic process were more abundant in the WT than in the mutant, whereas in ∆*blu7* genes belonging to the protein metabolic process were more abundant. The ∆*blu7* mutant lacked two GO biological process categories (Developmental process and Multicellular organismal process) among the induced genes compared to the WT, whereas the signaling process category was found as induced only in the ∆*blu7* mutant. In contrast, both the mutant and WT strain down regulated genes showed the same GO categories in biological process, however more genes constituted these categories in the case of the ∆*blu7* mutant (Fig. 2c). Even though the number of total light regulated genes in each strain is similar, the ∆*blu7* mutant has less than half of uniquely induced genes (27) of those found for the WT (67), and almost twice the number of unique down-regulated genes (82/50; Fig. 2d).

**Fig. 3:**
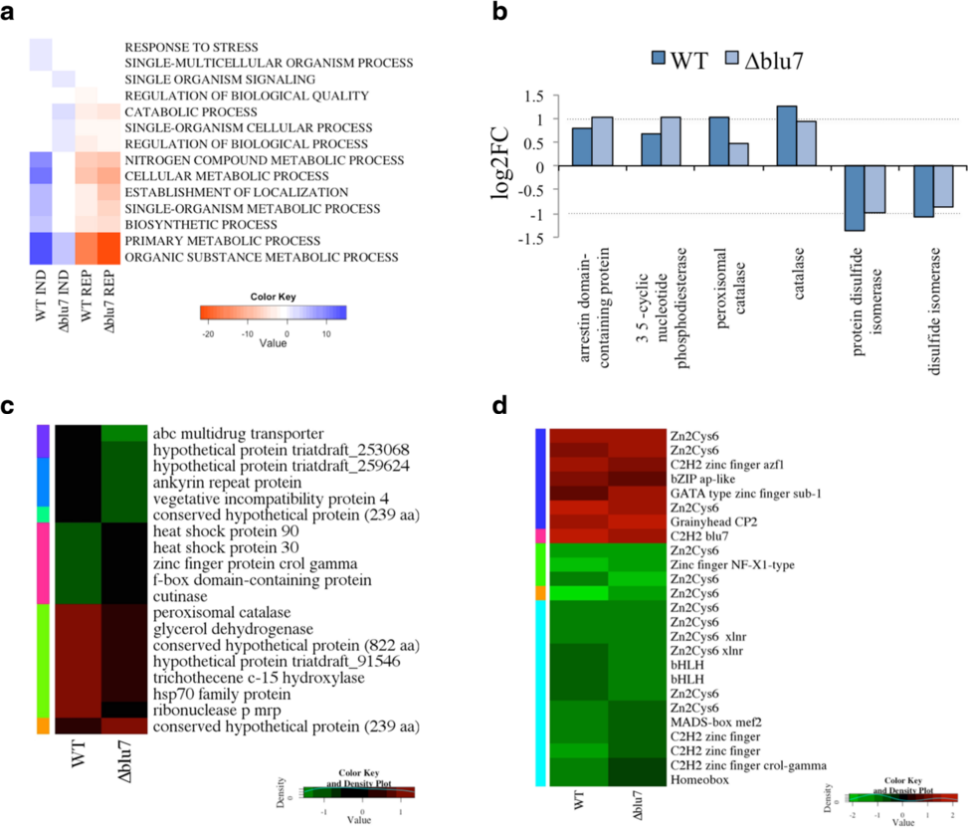
BLAST2GO annotation of the light regulated genes. **a** GO annotation by biological processes of the up- or down- regulated genes present only in WT or ∆*blu7*. **b** Transcriptional profile of selected genes from the transcriptome related to stress and signaling. **c** Light regulated genes present only in WT, not responsive in ∆*blu7* (1.5 > log2FC > −0.5), and light regulated genes present only in ∆*blu7*, not responsive in WT (1.5 > log2FC > −0.5) with hit description by BLAST2GO. **d** Heatmap of the transcription factors detected among the light regulated genes of both strains

BLAST2GO annotation by biological process showed that the response to stress, nitrogen metabolism, localization and biosynthetic process were up-regulated only in the WT (Fig. 3a), while processes related to single organism signaling and catabolic process were up-regulated only in the ∆*blu7* mutant (Fig. 3a). Although the same GO biological processes were present in the down-regulated genes of both strains (Fig. 3a), those genes down regulated by light only in the ∆*blu7* strain showed 12 repressed genes whose expression in the WT remained almost unchanged. Interestingly, more genes repressed only in ∆*blu7* mutant integrated the GO molecular process hydrolase activity, reported in *T. reesei* as regulated by the BLR proteins and Envoy [46]. The expression profile of the genes 138295 (arrestin binding domain) and 315387 (3-5’ cyclic phosphodiesterase), annotated in single organism signaling process, were induced only in the ∆*blu7* mutant. Those genes annotated as belonging to response to stress, were induced in the WT, and showed low expression levels in the mutant. Aside, genes related to correct protein folding were more strongly repressed in the WT (Fig. 3b). However the *hsp70* gene, also related to correct protein folding was induced only in the WT (Fig. 3c). Furthermore, we observed several genes encoding a ribonuclease p-mrp, an Hsp70 protein, a trichotecene c-15 hydrolase, a glycerol hydrolase, and a peroxisomal catalase with more than two fold induction in the WT that in the mutant did not show a change in expression after the light pulse (Additional file 8), making them potential targets of Blu7 regulation (Fig. 3c).

The molecular function of the most repressed genes (FC < 0.4) in the mutant or the WT strains was related to hydrolase activity, protein binding, transferase activity, and catalytic activity (Additional file 8).

Previous microarray studies in *T. atroviride* showed light induced expression of only one gene encoding a C2H2 zinc finger TF (*blu7*) [22]. Here, we found 25 TFs differentially regulated by light in both strains (Additional file 9). Twelve (48 %) belong to the Zn_2_Cys_6_ zinc finger family, five (20 %) to the C2H2 family, including Blu7, and the remaining 32 % to other families (Fig. 3d, Additional file 9). We detected six genes encoding TFs (Ids. 689, 288492, 53602, 173231, 164928, 234627; not studied yet) repressed more than 2 fold only in the WT (Additional file 9). On the other hand, we identified four transcription factor encoding genes repressed more than two fold only in ∆*blu7*.

### The Blu7 protein is involved in tolerance to continuous exposure to light

**Fig. 4:**
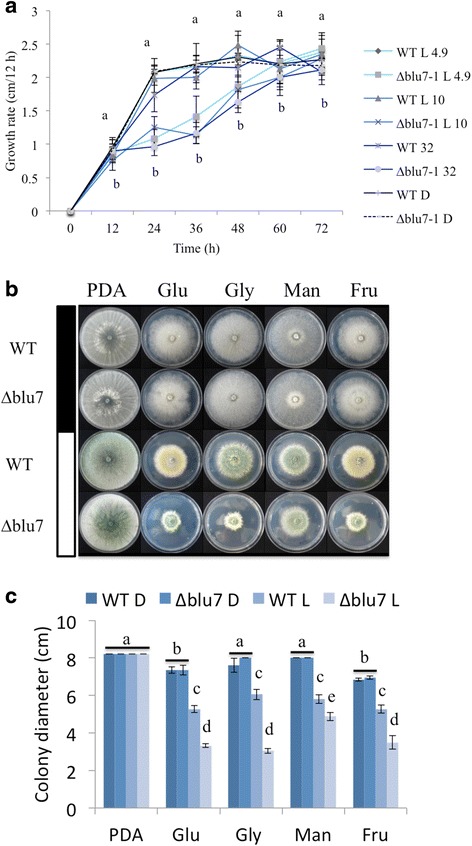
Reduced growth of the ∆*blu7* mutant cultivated under constant light. **a** Colony growth rate of the ∆*blu7* and WT strains registered every 12 h during 72 h when grown under exposure to 4.9, 10 or 32 μmolm^−2^s^−1^ blue-light on PDA. **b** Colonies of the WT and ∆*blu7* strains grown on minimal media with glucose (Glu), glycerol (Gly), mannitol (Man) or fructose (Fru) as a sole carbon source, PDA was used as control. Colonies were photographed after 72 h of growth in the carbon source indicated, grown either in the dark (filled bar) or under illumination with white light of 3.2 μmolm^−2^s^−1^ (empty bar). **c** Colony diameters measured after 72 h of growth. One-way ANOVA and pairwise *t*-test was applied to the data. Different letters indicate statistically significant differences. Three independent biological replicates were used for each strain

The reduction of colony growth of the ∆*blu7* mutant when grown under constant illumination prompted us to evaluate the response of the mutants and the WT under constant blue light. Radial growth and mycelial mass of the WT in darkness and constant exposure to blue light (4.9 μmolm^−2^s^−1^) was the same on PDA. However, a slight radial growth reduction at 10 and 32 μmolm^−2^s^−1^ of blue-light was noticed in the WT, although not statistically significant (Additional file 10). In contrast, the ∆*blu7* mutant strain showed a stronger growth reduction under constant blue light as fluence increased (Additional file 10). The growth delay of the mutant strain was observed already at 4.9 μmolm^−2^s^−1^ (mycelial mass: 0.150 ± 0.023 mg in the dark and 0.070 ± 0.012 mg in light), and was more evident at 10 and 32 μmolm^−2^s^−1^ of blue light (Fig. 4a). Growth inhibition by light in *T. atroviride* has been reported to be dependent on the BLR proteins and the carbon source available [35, 36]. Since the BLR complex induces *blu7* gene expression, we evaluated the growth of the mutant under constant white light using glucose, glycerol, mannitol or fructose as sole carbon sources on minimal media and on PDA (Fig. 4b). We observed growth inhibition under constant white light on all carbon sources tested for both the WT and the ∆*blu7* mutant strains (Fig. 4b). However, the growth reduction of the mutants on medium containing glucose, glycerol or fructose was 40 % more pronounced than that of the WT, whereas the growth rate on mannitol was similar to that of the WT (Fig. 4c). In this regard, it has been clearly established that primary and secondary metabolism processes are regulated by light in fungi [47, 48], but how are they regulated is still largely an open question.

**Fig. 5:**
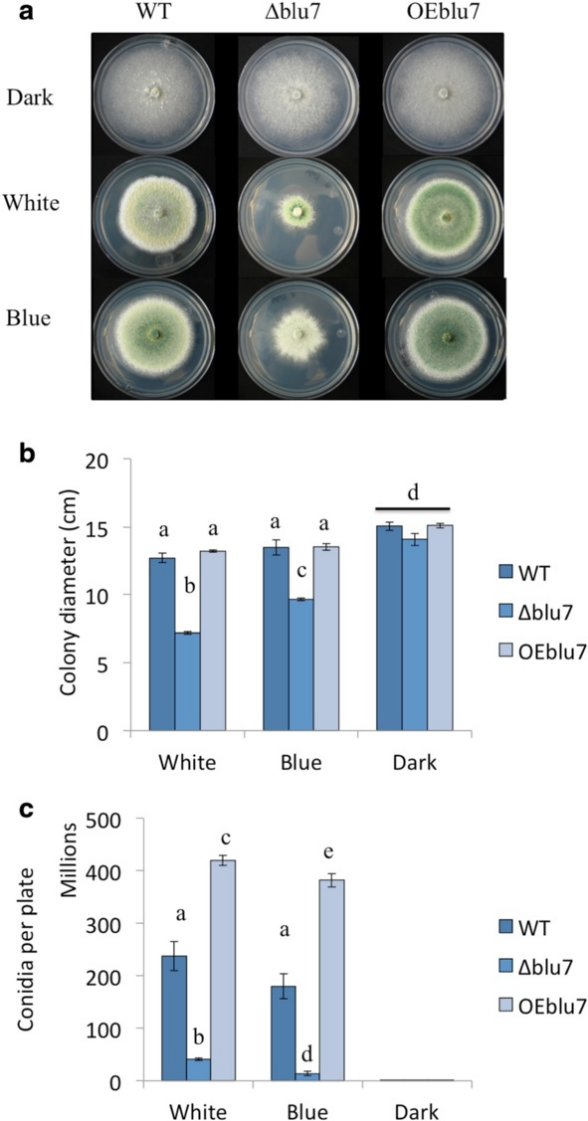
Effect of constant white and blue light effects on conidiation and growth of *T. atroviride* WT, ∆*blu7* and OEblu7 strains using glucose as sole carbon source. **a** Colony growth after 72 h of growth under constant white 3.2 μmolm^−2^s^−1^ or blue light 4.9 μmolm^−2^s^−1^ of the ∆*blu7*, WT and OEblu7 strains. **b** Colony diameter measured after 72 h. **c** Conidia production of the ∆*blu7*, WT and OEblu7 strains grown under constant white (3.2 μmolm^−2^s^−1^) or blue light (4.9 μmolm^−2^s^−1^). **b**, **c** One-way ANOVA and pairwise *t*-test was applied to the data. Different letters indicate statistically significant differences. Three independent biological replicates were used for each strain

To gain deeper understanding of the function of Blu7 in the light response, we obtained an overexpressing strain (OEblu7) of the *blu7* gene reported by Rosales et al. [22]. For this purpose the 642 bp CDS was placed under the control of the pyruvate kinase constitutive promoter. The 213 aa long overexpressed version of Blu7 (containing half of the polyQ, the pro-rich and the C2H2 zinc finger domain) was enough to increase more than twice conidiation after a pulse of blue light compared to WT levels (Additional file 11). Growth of the OEblu7 strain in darkness or in constant light (white or blue) did not differ from that of the WT on minimal media on glucose (Fig. 5a, b). However in constant light colonies of the OEblu7 strain appear to be greener, apparently due to higher production of conidia (Fig. 5a). Indeed, the OEblu7 strain produced 76 % more conidia than the WT under continuous exposure to white light, and 110 % more conidia when exposed to blue light (Fig. 5c), and no inhibition of growth by light was observed (Fig. 5a). The fact that the overexpressing transformant still required light to conidiate indicates that expression of *blu7* is not sufficient to trigger conidiation, and that other factors are required to achieve photoconidiation or that the Blu7 protein needs to be post-translationally modified in a light dependent manner.

Carbon starvation induces asexual reproduction in *T. atroviride* [34]. Interestingly, in the absence of the Blr proteins there is no response to this stimulus [19], suggesting that down stream targets of the Blr proteins regulate conidiation and growth under carbon starvation. The fact that growth of the ∆*blu7* mutants is reduced when compared to that of the WT, but only in the presence of light, indicates an alteration in their capacity to respond to light rather than a defect on carbon uptake. Consequently, we decided to analyze the transcriptional response of the ∆*blu7* mutant and the WT strain under constant light using glucose as sole carbon source to identify the genes that are deregulated and involved in growth under such conditions.

### Blu7 is involved in glucose metabolism under constant light

**Fig. 6:**
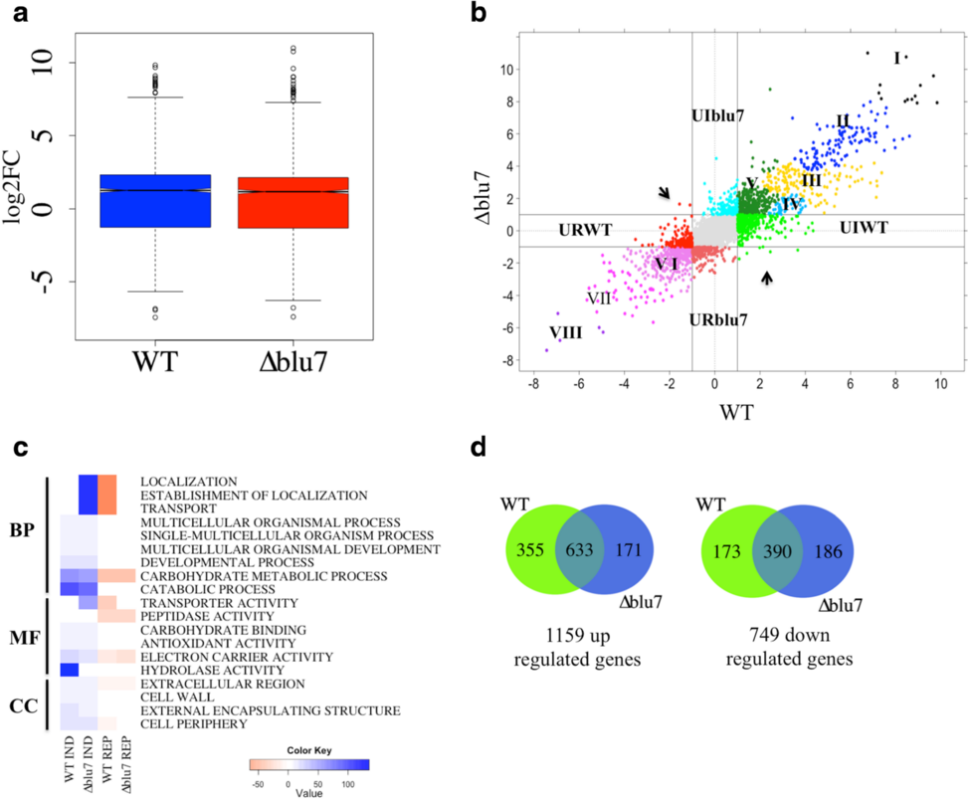
Comparison of the transcriptional response of the ∆*blu7* and WT strains under constant white light. **a** Boxplot of differential light regulated genes with |log2FC| > 1 for the ∆*blu7* and WT strains. **b** Overall plot of log2FC of the light regulated genes in WT vs ∆*blu7*. Hierarchical clustering results in 8 clusters (I, II, III, IV and V up-regulated and VI, VII and VIII for down-regulated). Horizontal lines of ±1 contain the genes differentially expressed only in the WT strain; vertical lines of ±1 contain the genes differentially expressed only in the ∆*blu7* mutant. **c** BLAST2GO annotation of the light regulated genes in WT or ∆*blu7* by biological process (PB), molecular function (MF) or cellular component (CC). **d** Venn diagram showing the overlap in up-regulated (left) and down-regulated (right) genes that respond in the WT and ∆*blu7* mutant strain

A ∆*blu7* mutant strain and the WT were cultivated under constant white light or in darkness with 2 % of glucose as sole carbon source during 60 h, and their transcriptome analyzed by RNAseq. Genes showing differential expression under these conditions may include direct targets of BLR, genes regulated by Blu7, and genes regulated by events taking place further downstream in the signaling cascade. Global expression of light regulated genes ranged from 891 to 0.005 FC for the WT, and between 1910 and 0.006 FC for the ∆*blu7* mutant (Fig. 6a). A total of 1901 light regulated genes were identified in both strains (Additional file 12), representing 16 % of the genome. In the WT strain, 1551 light regulated genes were identified and 1380 in ∆*blu7*, as compared to the corresponding controls grown in the dark. Interestingly we found five genes (Ids. 287033, 318140, 274363, 88516 and 267549) induced in WT but repressed in the mutant and two genes (Ids. 91844 and 323077) repressed in the WT but induced in the mutant. Twice as many uniquely induced genes were found in the WT (355) as in the mutant (171), and 633 genes overlapped between the two strains. In contrast, unique down-regulated genes were almost the same number in the WT (173) and the ∆*blu7* strain (186), and 390 common down-regulated genes were identified (Fig. 6d).

Hierarchical clustering of the light regulated genes results in eight main groups depicted by colors and roman numbers (Additional file 5B, Fig. 6b). In Cluster I (Additional file 12), 13 genes integrated the highly induced genes in both strains, which included the conidiation marker gene *con-10*, an orthologue of *N. crassa* [49]. Cluster II also has genes highly up-regulated in both strains, however 11 genes were more induced in the WT and 12 were more induced in the ∆*blu7* mutant (Additional file 12). Interestingly, the *env1* gene was more strongly induced in ∆*blu7* (FC = 142.3) than in the WT (FC = 57.3), suggesting an altered photoadaptation response. Previously reported light responsive genes of fungi were also found in this cluster, such as the orthologues of the *N. crassa clock controlled genes 1* and *6* (*ccg-1 & ccg-6*)*, and con-13*, showing similar induction levels in both strains [48–50]. In Cluster III more genes of the WT showed stronger induction, compared to that found in the *∆blu7* strain. GO-terms of those genes indicate that they are involved in metabolism (i.e., cytochrome, oxidoreductase, homoserine o-acetyltransferase), stress responses (i.e., *rds1* and *cry-DASH*) and transport (mfs, multidrug transporter genes). This cluster also encompasses genes that respond more strongly in the ∆*blu7* mutant than in the WT strain, two genes were involved in transport (mfs multidrug transporter, general transporter) and 5 genes were annotated in metabolism (aldehyde dehydrogenase, 2og-fe oxygenase family, glyconsiltransferase family, cytochrome, NAD dependent epimerase dehydratase encoding genes). Cluster IV contained genes more strongly induced in the WT than in the ∆*blu7* mutant (Additional file 11). In this case, the main GO terms enriched by biological process were related to biosynthetic, metabolic and nucleobase-containing compound metabolic process. In this cluster we found the orthologues of the *N. crassa clock-controlled gene 9* (*ccg-9;* Id. 77441) and *regulator of conidiation-1* (Id. 131307) induced to a higher extent in the WT than in ∆*blu7*. Additionally, we observed 14 genes in the WT with more than 3 fold induction compared to the ∆*blu7* mutant (Additional file 12). Cluster V (Additional file 5B, 11) contains genes with the lowest induction in the WT that in the ∆*blu7* mutant were more induced. The main genes in this group were annotated in transport and carbohydrate metabolic process. Mainly transporter encoding genes annotated as amino acid permeases and MFS multidrug transporters with more than two fold higher induction in the mutant.

Cluster VI contains slightly repressed genes under continuous illumination (0.5 > FC > 0.06). In this case, we observed 28 genes repressed more than two fold in ∆*blu7*; whereas in the WT strain, 89 of the genes detected showed twice stronger repression compared to the mutant. Genes more strongly repressed in ∆*blu7* were related mainly to metabolic process, and in the WT to metabolic process and transport. In general, cluster VII contains genes repressed to the same extent in the WT and the ∆*blu7* mutant. However we detected 16 genes with more than two fold repression in ∆*blu7*, two of them with peptidase activity (a serine endopeptidase and an alpha beta hydrolase) and the remaining genes were related to catalytic activity and hydrolase activity. In the case of the WT, we observed 13 genes repressed more than two fold, as compared to the mutant. In cluster VIII we found the five most repressed genes of the light regulated set. The benzoate 4-monooxygenase cytochrome P450 encoding gene was more strongly repressed in the WT (FC = 0.008) than in the mutant (FC = 0.02) and the cyclohexanone monooxygenase encoding gene (FC = 0.012) was more strongly repressed in the ∆*blu7* (FC = 0.012) compared to WT (FC = 0.03). While the tricothecene c15-hydroxylase and two cyclohexanone monooxygenase encoding genes were repressed to the same extent in the WT and the ∆*blu7* strains.

**Fig. 7:**
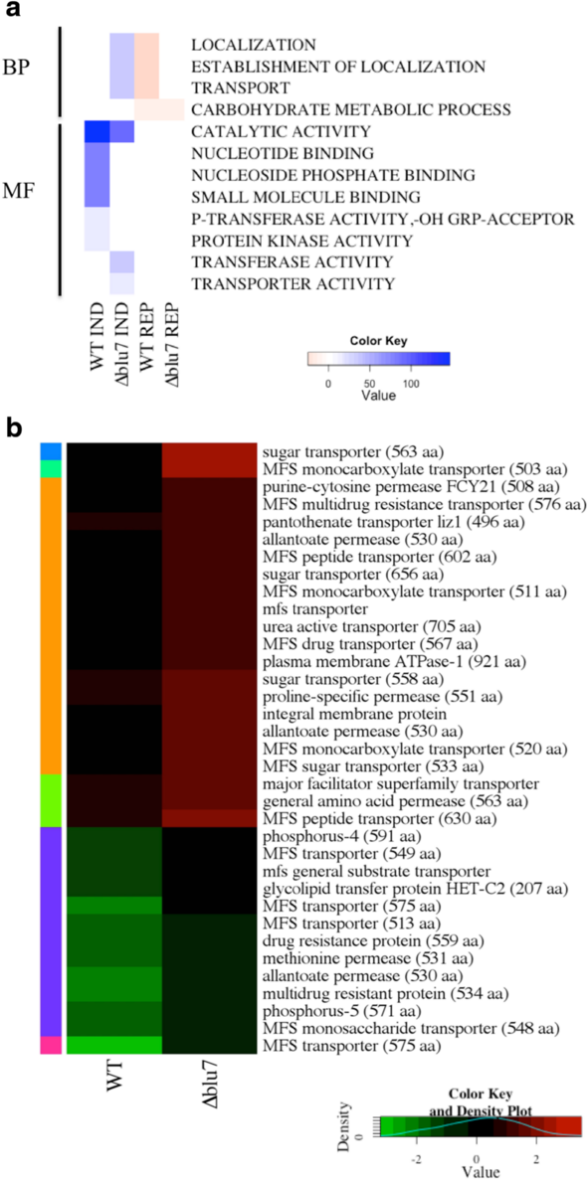
Analysis of genes differentially expressed only in the WT or the ∆*blu7* strains. **a** Enrichment analysis by biological process and molecular function of WT or ∆*blu7* mutant of unique light regulated genes. **b** Heatmap of annotated genes in localization, establishment of localization and transport process in the WT and the ∆*blu7* strains

**Fig. 8:**
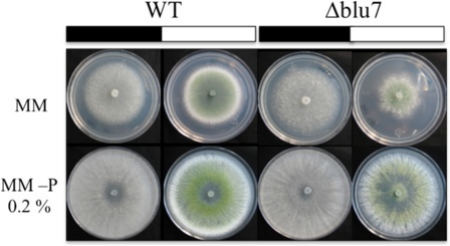
Growth under continuous exposure to light is rescued by a nitrogen rich medium. Growth of the wild type (WT) and ∆*blu7* mutant strain under constant white light (filled bars) or in the dark (empty bars), supplemented or not with 0.2 % of peptone, as indicated

Most of the GO-terms by Biological process (BP), Molecular function (MF) or Cellular component (CC) enriched in the up- or down- regulated genes were present in both strains (Fig. 6c). However, GO-terms enriched of the biological process categories related to Localization, Establishment of localization and transport were induced in the ∆*blu7* mutant but repressed in WT (Fig. 6c). Interestingly, we observed that the molecular function (MF) related to hydrolase activity (glycoside hydrolase activity family 76, 18, 15, 55, 54, 81, 18 and 47; as well as chitinases 3, 18–1 and endo-beta glucanases) was enriched only in the case of the WT induced genes. Furthermore, the term transporter activity was enriched in the WT repressed genes, and in the ∆*blu7* mutant induced ones. These data prompted us to analyze the genes that are light regulated only in one of the strains. Enrichment analysis of the unique up- or down- regulated genes in the WT or ∆*blu7* are shown in Fig. 7a. GO-terms: localization, establishment of localization and transport were enriched in down-regulated genes of the WT and enriched in the up-regulated genes of the ∆*blu7* mutant. We also observed GO-terms (small molecule binding, nucleoside phosphate binding, phosphotransferase activity, nucleotide binding and protein kinase activity) of induced genes enriched only in WT and GO-terms (transferase activity) of induced genes enriched only in the ∆*blu7* mutant. On the other hand, down-regulated genes were enriched in the GO-terms carbohydrate metabolic process and catalytic activity in both strains (Fig. 7a). Looking into transport and transporter activity, GO-terms enriched in the genes repressed in the WT and those induced in ∆*blu7*, we observed that these categories were mainly represented by general nitrogen transporter and sugar transporter encoding genes (Fig. 7b). Interestingly, the ∆*blu7* mutant presented general amino acid transporter and general monosaccharide transporter encoding genes as induced; whereas in the WT methionine transporter and general sugar transporter genes were repressed (Fig. 7b). These data indicate a nitrogen metabolism defect in the mutants, so we cultivated the WT and ∆*blu7* strains on minimal media supplemented with 0.2 % peptone under constant light during 60 h (Fig. 8). Radial growth of the WT and ∆*blu7* mutant was the same under constant light or in darkness when grown on peptone-supplemented media. However, production of conidia in the ∆*blu7* mutant was much lower than in the WT (Fig. 8), indicating that nitrogen sufficiency is enough to tolerate prolonged light exposure but not to support conidiation in ∆*blu7* mutants.

## Discussion

Asexual reproduction of *T. atroviride* is induced by light, nutrient limitation and mechanical damage. The BLR complex does not only regulate photoconidiation, but also conidiation in response to sudden carbon deprivation [34]. Notwithstanding, downstream components of the signaling pathway initiated through the BLR proteins are largely unknown. A cDNA microarray analysis identified a single putative transcription factor induced by a pulse of white light. Based on that microarray approach, covering only 12 % of the genes in the genome, 40 genes (2.8 %) were found to be light regulated. To extend our knowledge of the transcriptional response of *T. atroviride* to light to a genome wide scale, we used RNA-seq to analyze the role of the putative transcription factor Blu7 in the control of asexual development, light sensitivity, and response to light on glucose as carbon source.

The transcriptional response of the WT and ∆*blu7* mutant, 30 min after a pulse of 100 μmolm^-2^ of blue light, showed 4.8 % of the genome responsive with more than 2-fold change, similarly to *N. crassa, A. nidulans* and *A. fumigatus* [15, 26]. Interestingly we observed a significant correlation (R^2^ = 0.87) of the up-regulated genes shared between the Δ*blu7* mutant and the WT, in contrast the down-regulated genes were not correlated (R^2^ = 0.57), showing more negative values in log2FC of the ∆*blu7* responsive genes. These data indicate that Blu7 plays a particularly relevant role in tuning the regulation of light repressed genes.

The GO-terms (developmental and multicellular organismal process) induced after the pulse of light only in the WT were related to hypothetical genes, whereas in the signaling category present only in the *∆blu7* mutant the genes encoded Envoy1, a 3’–5’ cyclic nucleotide phosphodiesterase and an arrestin domain-containing protein. In this regard, it has been established that *env1* is an orthologue of the *N. crassa vivid* involved in the negative regulation of light responsive genes in a process called photoadaptation. While the cyclic nucleotide phosphodiesterase controls cAMP levels by hydrolyzing the phosphodiester bond in cAMP. In *Trichoderma* it is known that there is a transient biphasic oscillation in intracellular cAMP levels, activation of adenylyl cyclase, and phosphorylation of proteins upon exposure to a pulse of blue light. In fact, addition of exogenous cAMP to *Trichoderma* promotes sporulation even in the dark [51], whereas atropine, a compound known to inhibit adenylyl cyclase in *Neurospora* [52]*,* prevents sporulation even after photoinduction [53]. In addition, the *arrestin* gene*,* which encodes a protein that blocks coupling of the GPCR to G proteins, was more strongly induced in the mutant. Thus, changes in arrestin levels could contribute to lower cAMP production by impairing GPCRs GNA1 or GNA3 signaling. Furthermore, Envoy1 has been postulated as a regulator of cAMP levels in *T. reesei* trough repression of the cAMP phosphodiesterase [37], and the G-protein coupled receptors GNA1 and GNA3 direct that control of cAMP levels by regulating *env1* and their own expression. In agreement with our observations of the light induction of *blu7*, the corresponding mutants are less responsive to light, hence to conidiation, possibly due to reduced phosphodiesterase repression and partial loss of cAMP signaling of GNA1 or GNA3 by the presence of the Arrestin protein. Although, the cAMP accumulation in the ∆*blu7* mutant after the pulse of blue light requires further investigation in order to validate this hypothesis. Taking together these data, we suggest that in the *T. atroviride* WT strain cAMP might allow asexual reproduction after the pulse of light, but in the ∆*blu7* strain the missing signaling impairs conidiation at low light fluence. At high blue light fluence another level of regulation may exist, such as the accumulation of ROS or DNA damage, to induce conidiation in order to survive to extreme environments.

In agreement with these observations, a peroxisomal catalase and one heat shock protein were induced only in the WT, and another catalase was more strongly induced in the mutant than in the WT. Thus, the typical activation of stress responses observed in several fungi after exposure to light [54] is reduced in the *Trichoderma* ∆*blu7* mutants. It has been observed that oxidative stress stimulates differentiation in *N. crassa*, *Aspergillus* and *Trichoderma* [36, 55–57], and ROS produced upon light exposure induces conidiation in *N. crassa* [58]. In this sense, catalases and superoxide dismutases reduce ROS after the light pulse to re-establish cell homeostasis. Furthermore, it was recently shown that in *B. cinerea* the white collar proteins are required to deal with ROS produced under constant illumination, and that the Bcltf1 GATA TF, a homologue of SUB-1 of *N. crassa*, is important to cope with oxidative stress [59]. In the ∆*blu7* mutant described here we observed a higher expression of *sub-1,* which might, consequently result in lower levels of ROS after exposure to light, and might play a particularly relevant role at low light fluences.

Another possible explanation for the reduced catalases transcript levels and lower conidiation levels in the ∆*blu7* mutants is that there is a higher reducing power in these mutants than in the WT. In this regard, we observed 11 genes with oxidoreductase activity repressed only in the mutant. Also, we observed 6 genes encoding enzymes with reductase activity (cyclohexanone monooxygenase, lipooxigenase 1, berberin family protein, FAD monooxygenase, FAD binding domain protein, NADPH dehydrogenase) more strongly induced in ∆*blu7* and 4 genes (encoding: short chain dehydrogenase reductase, oxidoreductase protein, FAD dependent oxidoreductase, NADH-flavin oxidoreductase NADH oxidase family protein) repressed only in the WT. In addition, there were 2 genes (encoding cyclohexanone monooxygenase, & FAD monooxygenase) repressed to a lower extent in the ∆*blu7* mutant (FC 0.49, 0.47) than in the WT strain (FC 0.36, 0.36). Therefore, the reduced levels of ROS signaling and cAMP production would not be sufficient to trigger conidiation in the ∆*blu7* mutants at low light fluence. This defect might be compensated at high light fluence by higher production of ROS, although under those conditions these signals might be regulated through another mechanism.

In *N. crassa* and *A. nidulans* the WCC controls several transcription factors that in turn regulate asexual reproduction [15, 17, 26, 60]. Among the transcription factors regulated by a pulse of blue light only 6 have GO annotation. We observed two TF (*cp2* and *sub-1*) more strongly induced in the ∆*blu7* mutant. GRHY-like or CP2 has been found in several fungal transcriptomes of light treatments [15, 24, 26]. Recently a CP2/GRHY-like was identified as a conidial separation-2 (*csp-2*) allele in *N. crassa* and shown to be involved in conidial separation and spore release by cell wall remodeling [39]. Reports on the study of a *sub-1* deletion mutant showed that its presence is required to activate late light regulated genes in *N. crassa*. However, the mutant presents defects only in sexual development [15, 25]. It might be that some of the genes more strongly induced in the ∆*blu7* mutant are not directly under its control, but at least are in part regulated by CP2 or SUB-1.

Under constant illumination, we observed a growth delay in the ∆*blu7* mutant at high light intensities (above 10 μmolm^−2^s^−1^). This is in agreement with the requirement of the BLR proteins to grow in the presence of blue or red light in *T. atroviride* [19]. Under constant illumination the BLRC must regulate genes to deal with the constant exposure to light (i.e., growth, morphogenesis, stress). The growth delay of the mutants under constant blue light suggests that Blu7 participates in light tolerance. Reduced tolerance of the *∆blu7* mutants was much more evident when glucose was used as sole carbon source compared to rich media, such as PDA, suggesting a role of Blu7 in carbon metabolism to tolerate continuous light exposure. The combination of light with different carbon sources differentially stimulates conidiation and growth, and this response depends on the BLR proteins [35, 36]. In addition, the downstream target of the BLRC, Envoy1 of *T. reesei* is involved in carbon dependent growth in the presence of light.

Since light only temporarily delayed growth of the mutants, we suggest that they are affected in their capacity to tolerate light. These phenotypes resemble those of the ∆*env1* mutants of *T. reesei* and *T. atroviride* in constant light on PDA [61, 62]. In the *env*1 mutants of *T. reesei* the lack of the negative feedback loop, over the light regulated genes controlled by the BLRC, leads to a reduced growth [61, 62]. In this loop Envoy acts as a repressor of negative regulators of growth activated by the BLR proteins. Accordingly, the stronger induction of *env1* in ∆*blu7* suggests that Blu7 participates as a positive regulator of growth in this negative feedback loop; however, further investigation is required to test this hypothesis.

Conidiation of the ∆*blu7* mutant was not completely impaired but reduced in constant light. This is also in agreement with the phenotype of ∆*env1* mutants of *T. atroviride* which produce more conidia than the WT strain in response to light, likely due to a longer permanence of mRNAs of photoconidiation genes, such as *blu7* [62]. In the ∆*blu7* mutant, the increased induction of *env1* may lead to fast shut down of the photoconidiation genes and the absence of Blu7 in reduced cAMP levels, due to increased transcript levels of the cAMP phosphodiesterase gene, and thereof of its enzymatic activity.

The differentially expressed genes observed in the ∆*blu7* and the WT grown on glucose containing medium under continuous exposure to white light reflected major metabolic changes in both strains compared to darkness. Since in darkness, growth of the WT and the *∆blu7* mutant was similar, glucose uptake or metabolism is not deficient or impaired. This points to a light activation of the Blu7 protein or its light regulation through an interacting protein to support metabolic changes. Interestingly, GO-terms related to localization and transport categories were repressed in the WT and induced in the ∆*blu7* mutant. These categories contained genes encoding sugar, amino acid and multidrug transporters. In addition, we observed two-fold higher induction of the *nmrA* gene (Id 35890) in the WT (FC = 3.9) compared to the *∆blu7* mutant (FC = 1.8). In *A. nidulans*, NmrA is a negative regulator of nitrogen metabolite repression, which controls expression of enzymes and permeases necessary for the use of non-preferred nitrogen sources [63, 64]. Thus, the partial nitrogen metabolism deregulation observed in the ∆*blu7* mutant might be due to *nmrA* repression, leading to the induction of several amino acid transporter genes in *∆blu7*. In *M. oryzae* the *tps1* gene (encoding a trehalose-6-phosphate synthase), a glucose-6-phosphate sensor, negatively regulates NMR inactivating Nmr1-3. Also the multidrug and toxin extrusion (MATE)-family pump Mdt1, a downstream target of Tps1, is involved in glucose assimilation, conidiation and virulence [65]. Similarly, we observed several monosaccharide and general sugar transporters induced in ∆*blu7* and repressed in the WT, as well as multidrug transporters induced in both strains. These observations point to the light regulation of carbon and nitrogen metabolism to stimulate conidiation and growth in *T. atroviride*, respectively.

Carbon or nitrogen starvation results in the formation of less branched compact hyphae than those observed in rich media, in addition the sudden lack of nitrogen or carbon source in *T. atroviride* resembles the ring of conidiation triggered by a pulse of light [19]. Interestingly, in the absence of the *blr1* or *blr2* genes the induction of conidiation by carbon (glucose) starvation is lost, in contrast nitrogen starvation can still stimulate conidiation, suggesting an independent regulation of asexual reproduction by the BLR proteins [34]. Thus we hypothesized that nitrogen metabolism should be involved in hyphal growth under constant light. Several genes encoding enzymes with peptidase activity (papain cysteine protease, candidapesin-3 precursor, family a1 protease, microbial serine protease, extracellular alkaline serine protease), including an intracellular serine protease, were induced in the WT strain, suggesting that protein degradation or recycling of nitrogen resources is activated in response to light. Nitrogen metabolism related genes have also been found among the light regulated genes of *N. crassa* [15, 23].

In accordance with these observations, we found an allantoinase-encoding gene (Id. 300514), involved in purine metabolism, induced only in the WT. Besides two allantoate permeases (Ids. 257840 & 127784) were repressed in the WT, but not in the ∆*blu7* strain, and two allantoate transporters (Ids. 302043 & 35866) were induced only in the mutant - allantoate is used as nitrogen storage in plants [66]. Transport of amino acids was repressed in the WT, mainly methionine transport. In contrast, in ∆*blu7* higher induction levels of genes encoding amino acid transporters and allantoate transporters were observed. These data are highly coincident with the transcriptional response to nitrogen starvation reported in the phytopathogenic fungus *Magnaporthe grisea* [67]. Taken together, our data appear to indicate that the absence of Blu7 mimics a nitrogen starvation condition when *Trichoderma* is grown under constant exposure to light, which results in clearly reduced growth. Restoration of growth rate and radial colony size of the WT and ∆*blu7* mutants by the addition of peptone to the media suggests that light growth inhibition is mainly due to nitrogen intracellular metabolism, and that Blu7 indirectly participates in the regulation of nitrogen metabolism in the presence of light under limited nitrogen supply (Fig. 8). As a consequence of the lack of Blu7, the mutants have lower capacity to recycle nitrogen sources, requiring the induction of general amino acid transporters, which in the presence of peptides from peptone are taken up and metabolized, resulting in much better growth.

We also observed misregulation of the ras GTPase (*rsr1;* Id. 300901), which was induced to higher levels in the ∆*blu7* (FC = 5.2) mutant than in the WT (FC = 2.0). Activation of this pathway in yeast involves a G-protein coupled receptor and RAS signaling to regulate glucose availability [68, 69]. From the GPCRs regulated in both strains only the predicted GPCR gene Id. 40423 is more strongly induced in the ∆*blu7* mutant, which might act as an upstream regulator. GPCRs are involved in amino acid and carbon source sensing as well as in cAMP perception. The *gpr1* gene (Id. 83166), encoding a homolog of the *N. crassa* cAMP receptor Gpr1 [70, 71], is induced only in the WT strain. Silencing of *gpr1* in *T. atroviride* P1 reduces growth, conidiation and secondary metabolism [71]. In Yeast the GPR1-GPA2 GPCR system is involved in the regulation of cAMP signaling [69]. The absence of Gpr1 in the ∆*blu7* mutants in conjunction with the increase in cAMP phosphodiesterase might be reflected in the lack of protein kinase A (PKA) activity resulting in the loss of this signaling pathway to regulate the light response. PKA signaling after a pulse of light is required to regulate up or down responsive genes, also regulated by the BLR proteins [19, 34]. We further observed 6 serine-threonine protein kinase encoding genes induced only in the WT, which might exert posttranslational modifications in transcription factors, consequently affecting regulation of gene expression in response to light, or might directly affect enzyme activity, resulting in altered metabolism.

**Fig. 9:**
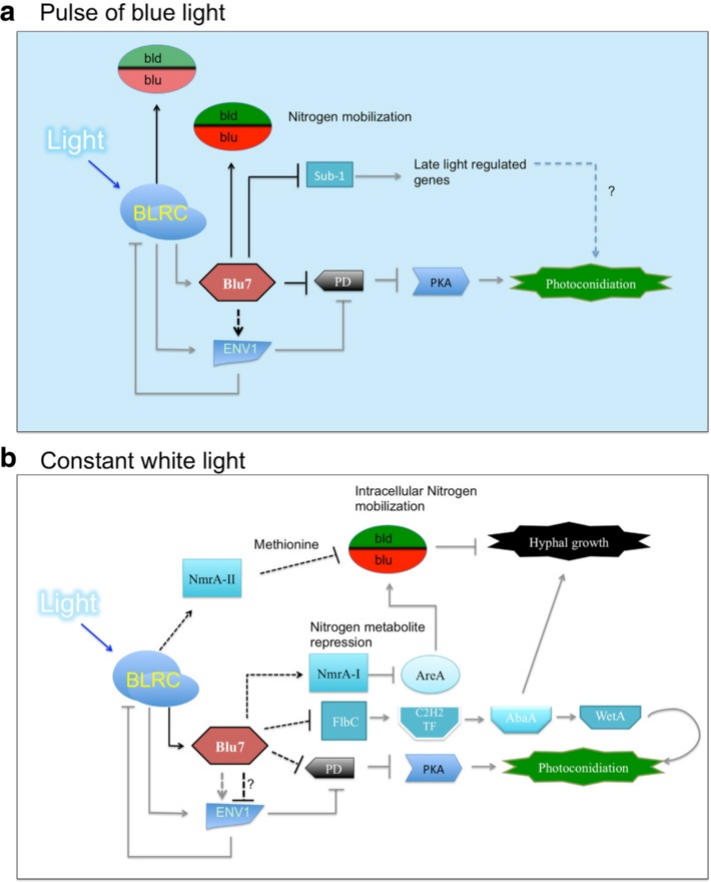
Model of the role of the transcriptional regulator Blu7 in the molecular response of *T. atroviride* to light. **a** Molecular responses to a pulse of blue light. Exposure of *T. atroviride* to low fluence blue light perceived by the BLRC results in the transcriptional activation of *blu7*, *sub1*, *azf1*, *xln*r and 20 other TF encoding genes, as well as the induction of Envoy. Blu7 tunes photoconidiation by repressing the expression of the 5’-3’ cAMP phosphodiesterase encoding gene (PD). Likewise, the induction of *env1* enhances PD repression, while transitory accumulation of cAMP activates PKA that in turn regulates photoconidiation genes [34]. Nitrogen mobilization (amino acid transporters) is indirectly regulated by Blu7 and modulates the balance between conidiation and mycelial growth. **b** Molecular responses to continuous light exposure. Under constant white light, *blu*7 induction by the BLRC is regulated by the carbon source available in the medium. ENV1 negatively regulates BLRC target genes for adaptation to constant light. When *T. atroviride* is grown on glucose containing medium, *blu7* limits the induction of *env1*. Blu7 is required for nitrogen mobilization through the inhibition of the orthologue of AreA by the negative regulator NmrA up-regulation. In addition, hyphal growth and conidiation are tightly regulated by the amount of FlbC to induce the sequential expression of the transcription factors C2H2 ➜AbaA ➜ WetA. Black lines indicate control points supported by our RNA-seq data and gray lines are based on previously reported experimental evidence

An overview of the data presented here is depicted in Fig. 9. The BLRC perceives light and induces *blu7* expression. Transient increase of cAMP by inhibition of the 3’–5’ cAMP phosphodiesterase leads to activation of photoconidiation genes by PKA. Growth rate under constant light depends on nitrogen availability, controlling the uptake from the media or mobilization of stored nitrogen (Fig. 9).

## Conclusions

The present work describes the transcriptional response to light of the mutant in the putative transcription factor encoding gene *blu7*, a BLRC dependent light regulated gene in *T. atroviride*. The *blu7* gene is required for photoconidiation at low but not at high blue light fluence. The absence of *blu7* resulted in increased levels of expression of the 3’-5’ cAMP phosphodiesterase encoding gene, which could explain the reduced conidiation observed in the mutant*.* Furthermore, the increased expression of *env1* observed in the ∆*blu7* strain under constant illumination suggests linked roles in photoadaptation between these two regulators. Interestingly, the growth inhibition response of the *blu7* replacement mutant to light was reduced in rich media. The decreased expression of energy and metabolism related genes in the ∆*blu7* mutant under constant light may explain the slower growth, rendered by defective nitrogen metabolism.

### Ethics (and consent to participate)

Not applicable.

### Consent to publish

Not applicable.

## Methods

### Strains and culture media

All *Trichoderma* strains were cultivated on potato dextrose agar (PDA) at 28 °C under the light regime indicated. The wild type strain used was IMI206040. The ∆*blu7* mutant was obtained by replacement of 664 bp of the gene locus of the WT strain IMI206040. An overexpressing version of *blu7* was generated using the *pki* (pyruvate kinase) constitutive promoter inserted in plasmid pUE10 [31]. Culture minimal medium (MM) contains K_2_HPO_4_ 5.1 mM, MgSO_4_ 1.7 mM, KCl 2.7 mM, NH_4_NO_3_ 1.25 mM, FeSO_4_ 13.1 μM, ZnSO_4_ 7 μM and MnCl_2_ 7.9 μM, and 2 % glucose as carbon source, unless otherwise indicated, and 1.5 % bacto-agar, and was adjusted to pH 4.8. Constant white light treatments were carried out with a fluorescent lamp at a 4.9 μmolm^−2^s^−1^ fluence. Blue light treatments were carried out in a chamber equipped with light emitting diodes (LED). All other manipulations were carried out in the dark using only a safety red light with 0.5 μmolm^−2^s^−1^ fluence.

### RNA and DNA manipulations

The *blu7* cDNA sequence was initially obtained from a standard cDNA library generated from polyA^+^ RNA obtained from blue-light induced mycelia of *T. atroviride*. Since this cDNA appeared to be incomplete, we walked on the transcript using RT-PCR reactions and primers at different positions up-stream of the original transcript, aided by the genome sequence. The complete cDNA clone sequence was deposited in the GeneBank (Id KU666056). This sequence was supported by our Illumina transcriptome data (see below).

Gene replacement ∆*blu7* constructs were obtained by the double-joint PCR method [38]. The first PCR was made using the primers Pblu7-F, PQblu7-R and TQblu7-F, Tblu7-R (Additional file 13) to amplify 1.4 kb fragments up- and down-stream of the 664 bp fragment to be replaced respectively, and joined them to 1.4 kb of the hygromycin phosphotransferase resistance cassette (hph), amplified by PCR from plasmid pCB1004 using the primers hph-F and hph-R (Additional file 13), in a second PCR without primers. Finally, the nested primers N5’-BLU7-F and N3’-BLU7-R were used in a third PCR reaction to amplify the 4.2 kb construct containing the joined 5’UTRblu7-hph-3’UTRblu7 fragments. Transformants overexpressing *blu7* were obtained using a construct made by amplifying the cDNA of *blu7* with the primers ORFBLU7-F and ORFBLU7-R and cloning it in TOPO-PCR 2.1. The plasmid was then digested with *Eco*RI and *Bam*HI, and the cDNA fragment inserted into *Eco*RI and *Bam*HI sites of pUE10 [31]. The resulting plasmid carries the *blu7* cDNA under the control of the constitutive promoter of the pyruvate kinase gene from *T. reesei,* and 1.4 kb of the 3’ UTR of the *blu17* gene of *T. atroviride*. Both constructions were directly used for PEG-mediated protoplast transformation of the WT strain as previously described [38]. After three rounds of single spore isolation, fungal DNA from the WT, ∆*blu7* and OEblu7 strains was obtained according to the protocol reported of Raeder and Broda [72]. Southern blot analysis of the ∆*blu7* mutants was carried out digesting genomic DNA with *Xho*I, using as probe a 1.4 kb of the terminator region of *blu7*. For RNA preparation, mycelia were scraped from the surface of cellophane under red safelight (0.05 μmolm^−2^s^−1^), immediately frozen in liquid nitrogen, and RNA extracted with TRIzol according to the manufacturer recommendations (Invitrogen, GIBCO-BLR).

### Light treatments of ∆*blu7* mutants

Light-response curves of WT and ∆*blu7* mutants to blue light fluence were carried out in a growth chamber equipped with light-emitting diodes (Percival Scientific, Wisconsin, U.S.A.). A plug of mycelium from a colony grown on PDA for 48 h in darkness was used as inoculum in all experiments. Inocula of all strains (WT, *∆blu7*, OEblu7), for photoconidiation experiments, were cultivated in darkness for 36 h before exposure to a pulse of blue light of 50 (2.9 μmolm^−2^s^−1^, 17.2 s), 80 (2.9 μmolm^−2^s^−1^, 27.6 s), 100 (2.9 μmolm^−2^s^−1^, 34.5 s), 150 (6.8 μmolm^−2^s^−1^, 22 s), 250 (6.8 μmolm^−2^s^−1^, 30.7 s), 500 (10.35 μmolm^−2^s^−1^, 34.5 s), 1200 (28.2 μmolm^−2^s^−1^, 42.5 s), 2400 (30.7 μmolm^−2^s^−1^, 78.2 s), 4800 (30.7 μmolm^−2^s^−1^, 156.4 s), 9600 (30.7 μmolm^−2^s^−1^, 312.7 s) and 11000 (30.7 μmolm^−2^s^−1^, 358.3 s) μmolm^−2^, and then placed back in the dark for 36 h. Conidia were collected from plates with a micropipette upon addition of 2 ml sterile water to the plate and carefully scratching the surface of the colony with a sterile metal rod. Conidia from three independent biological replicates for each strain were then quantified using a hematocytometer.

For gene expression analysis of the *blu7, env1, sah1* and *gpd* genes after a pulse of light, *T. atroviride* WT or *∆blu7* mutant strain were exposed to 100 μmolm^−2^ (2.9 μmolm^−2^s^−1^, 34.5 s) of blue light using three biological replicates. After the blue light pulse, the mycelia were incubated in the dark for 30 min and collected for RNA extraction. Cultures of the WT or ∆*blu7* strains maintained always in the dark were used as controls of gene expression. Semiquantitative RT-PCR analyses were performed with superscript II transcriptase and Recombinant polymerase kits from Invitrogen. PCR’s were carried out in a final volume of 25 μl with the protocol specified by the manufacturer. To obtain the cDNA, 2 μg of total RNA were used from each condition using reverse specific primers for each gene evaluated. The pairs of primers (Additional file 13) used were qBlu7-F – qBlu7-R for *blu7*, SAH1RT-5 F – SAH1RT-3R for *sah1*, ENV-F – ENV-R for *env1* and GPD-F – GDP-R for *gpd*. The PCR condition was 95 °C, 3 min; 20 cycles of 95 °C, 20 s; 60 °C, 20 s and 72 °C, 20 s; and a 1 min final extension at 72 °C.

Growth assays under constant blue light of 4.9, 10 and 32 μmolm^−2^s^−1^ were carried out using a light emitting diodes (LED) equipped chamber at 28 °C during 72 h. Radial growth of the WT and ∆*blu7* mutant strains was determined every 12 h.

To evaluate the impact of continuous exposure to light on growth with carbon limitation, the WT and *∆blu7* mutant strains were cultivated in minimal media with 2 % glucose, glycerol, mannitol or fructose under a 4.9 μmolm^−2^s^−1^ white light at 28 °C during 72 h.

Mycelium of the WT strain (IMI206040) and the *∆blu7* mutant collected after 60 h of growth under constant white light (4.9 μmolm^−2^s^−1^) on glucose as sole carbon source was used for RNA-seq. Cultures of the WT and *∆blu7* mutant maintained always in the dark during 60 h were used as controls for gene expression analyses. All experiments were carried out using three biological replicates for each strain in different days.

All RNA samples (Blue light pulse or continuous illumination) were processed and subjected to high-throughput sequencing using an Illumina HiSeq^TN^2500 in the core facilities of Cinvestav (Irapuato, Guanajuato, Mexico).

### Analysis of the transcriptome data

The Illumina sequencing data was grouped in 6 reads libraries of each light treatment for WT or ∆*blu7* mutant, 3 read libraries for the dark control and 3 for the light treatment, derived from three biological replicates carried out in independent days, starting from different pre-inoculum. Read libraries were mapped to the genome of *T. atroviride* using bowtie2 version 2.0.0-beta7 with very sensitive default parameters [73, 74]. Reads counts were normalized by counts per million in the R version 2.15.2 environment using EDGER [75]. The same program was used to obtain differentially expressed genes using a cut-off of two-fold change and 0.01 of FDR between samples. Protein sequences in FASTA format was obtained from the Frozen gene catalog of *T. atroviride* genome V2 (http://genome.jgi.doe.gov/Triat2/Triat2.download.html). The corresponding proteins were compared against the MIPS database with a cutoff of 1x 10^−3^. GO term assignation was performed using an E-value ≤ 1 x10^−3^, an annotation score ≤ 40, a GO weight of 5 [76]. Additionally, we used BLAST2GO with an E-value < 1x10^−3^ for the annotation of all light regulated genes [77]. The differentially regulated genes associated proteins were annotated and showed as multilevel graphs by biological process, molecular function and cellular component, the representative level with more than 5 components was showed. GO enrichment analysis for each set of differentially expressed genes was performed using as reference the normalized set of genes with evidence of expression with at least two counts in one of the analyzed condition and with at least six counts among all conditions. In this analysis, Blast2GO [77] was used to compute enriched GO terms applying Fisher’s exact test as implemented in GOSSIP [76, 78]. GO terms with 0.05 FDR and q-value ≤ 0.05 were considered as significantly enriched in each comparison.

### Availability of data and materials

RNA-Seq were deposited in the NCBI BioSample database (http://www.ncbi.nlm.nih.gov/biosample/) with the IDs SAMN04022924 and SAMN04025729, and linked to the sequence read archive (SRA, http://www.ncbi.nlm.nih.gov/sra) with the following IDs: SRS1050830 and SRS1051774. The sequence of the *blu7* cDNA can be found in the GeneBank with accession number BankIt1891430 Seq1 KU666056.

## Additional files

Additional file 1: Figure S1. A) Overview of 4500 bp of the *blu7* gene locus showing the reads mapping to this region. The JGI Genwise predicted structure for the *blu7* gene (ID 284873) and that derived from our cDNA clone are schematically represented. B) Schematic representation of the domains found for the Blu7 deduced protein. The scheme indicates the proline rich region (PP, green box), the putative activation domain (QQQ, red box), the nuclear localization signal (NLS, light grey box) and the C2H2 zinc finger domain (ZnF C2H2). Numbers indicate amino acid positions. The dotted box shows the sequence deleted as a result of the gene replacement event. (JPG 1968 kb) Additional file 2: Figure S2. Southern blot analysis of *blu7* mutants. A) Schematic representation of the gene replacement event. Blue and red boxes represent the *blu7* ORF and its 5’ & 3’ flanking regions, respectively; and restriction sites for *Xho*I, the enzyme used for DNA digestion indicated. The genes replacement construct is represented by the hygromycin resistance cassette (hph; green box), and the *blu*7 5’ & 3’ flanking regions (red boxes). The probe, a 1.4 kb-long segment corresponding to the 3’ UTR of the *blu7* locus, is also indicated. B) Autoradiogram of the Southern analysis. The expected 2.7 kb signal for the WT and the 4.6 kb for the ∆*blu7* is shown due to a loss of the *Xho*I site by the *hph* replacement. (JPG 873 kb) Additional file 3: Figure S3. Light growth response of the WT and ∆*blu7* strains under constant illumination. A) Growth rate of WT and ∆*blu7* mutant during 72 h in darkness (D) or constant white light. B) Growth rate of WT and ∆*blu7* mutant during 72 h in constant blue light. C) Growth rate of the ∆*blu7* mutant and WT under photoperiod of blue light- dark conditions during 72 h. D) Conidia production of the WT and ∆*blu7* mutant under constant white (3.2 μmolm^−2^s^−1^) or blue (4.9 μmolm^−2^s^−1^) light treatments. One-way ANOVA and a pairwise *t*-test were applied to 6 independent replicates; asterisks indicate statistically significant differences (α < 0.05). (JPG 1758 kb) Additional file 4: Table S1. List of responsive genes in WT or ∆*blu7* 30 min after a pulse of 100 μmol of blue light compared to dark control of each strain. (CSV 77 kb) Additional file 5: Figure S4. Heatmap of the overall light regulated genes of WT and ∆*blu7* mutant. Hierarchical clustering of the differential genes after a pulse of 100 μmolm^−2^ of blue light (A) or under constant white light (B) of the WT and ∆*blu7* mutant is shown. (JPG 1801 kb) Additional file 6: Table S2. List of the most induced genes 30 min after a pulse of 100 μmolm^−2^ of blue light shared in the mutant and the WT strain. (CSV 9 kb) Additional file 7: Table S3. List of the most repressed genes after 30 min of 100 μmolm^−2^ of blue light shared in the mutant and the WT strain. (CSV 14 kb) Additional file 8: Table S4. List of the most responsive genes present only in the WT or the ∆*blu7* mutant strains. (CSV 9 kb) Additional file 9: Table S5. List of putative light responsive transcription factors. All putative transcription factors responding 30 min after a 100 μmolm^−2^ pulse of blue light in the WT or ∆*blu7* mutant are listed. (CSV 5 kb) Additional file 10: Figure S4. A) Phenotype of the WT and ∆*blu7* after under constant blue light of 4.9, 10 and 32 μmolm^−2^s^−1^. B) Radial colony growth after 72 h of light treatment of the WT and ∆*blu7* strains. One-way ANOVA and a pairwise *t*-test were applied to data. Different letters indicate statistically significant differences (α < 0.05). (JPG 1062 kb) Additional file 11: Figure S5. Stimulation of photoconidiation by several blue light fluences (from 50 to 11000 μmolm^−2^) in the WT, ∆*blu7* mutant and overexpressing (OEblu7) strains. (JPG 1487 kb) Additional file 12: Table S6. List of genes responding after 60 h exposure to constant white light on glucose as carbon source compared to dark control in the WT or ∆*blu7* strains. (CSV 257 kb) Additional file 13: Table S7. List of primers used in this study. Primers used to obtain the ∆*blu7* mutant and OEblu7 strains, as well as the primers used in RT-PCRs are listed. (CSV 1 kb)

## Abbreviations

bld: blue light down-regulated
BLR: blue light regulator
blu: blue light up-regulated
cAMP: cyclic adenosine monophosphate
FC: fold change
FDR: false discovery rate
GO: gene ontology
PD: phosphodiesterase
RNA-seq: ribonucleic acid sequencing
TF: transcription factor
WC: white collar
WT: wild type
