# Supplementary figures and images for: The Trichoderma atroviride putative transcription factor Blu7 controls light responsiveness and tolerance
Source: BMC Genomics. 2016 May 4;17:327. doi: 10.1186/s12864-016-2639-9 (PMC4855978; doi:10.1186/s12864-016-2639-9)

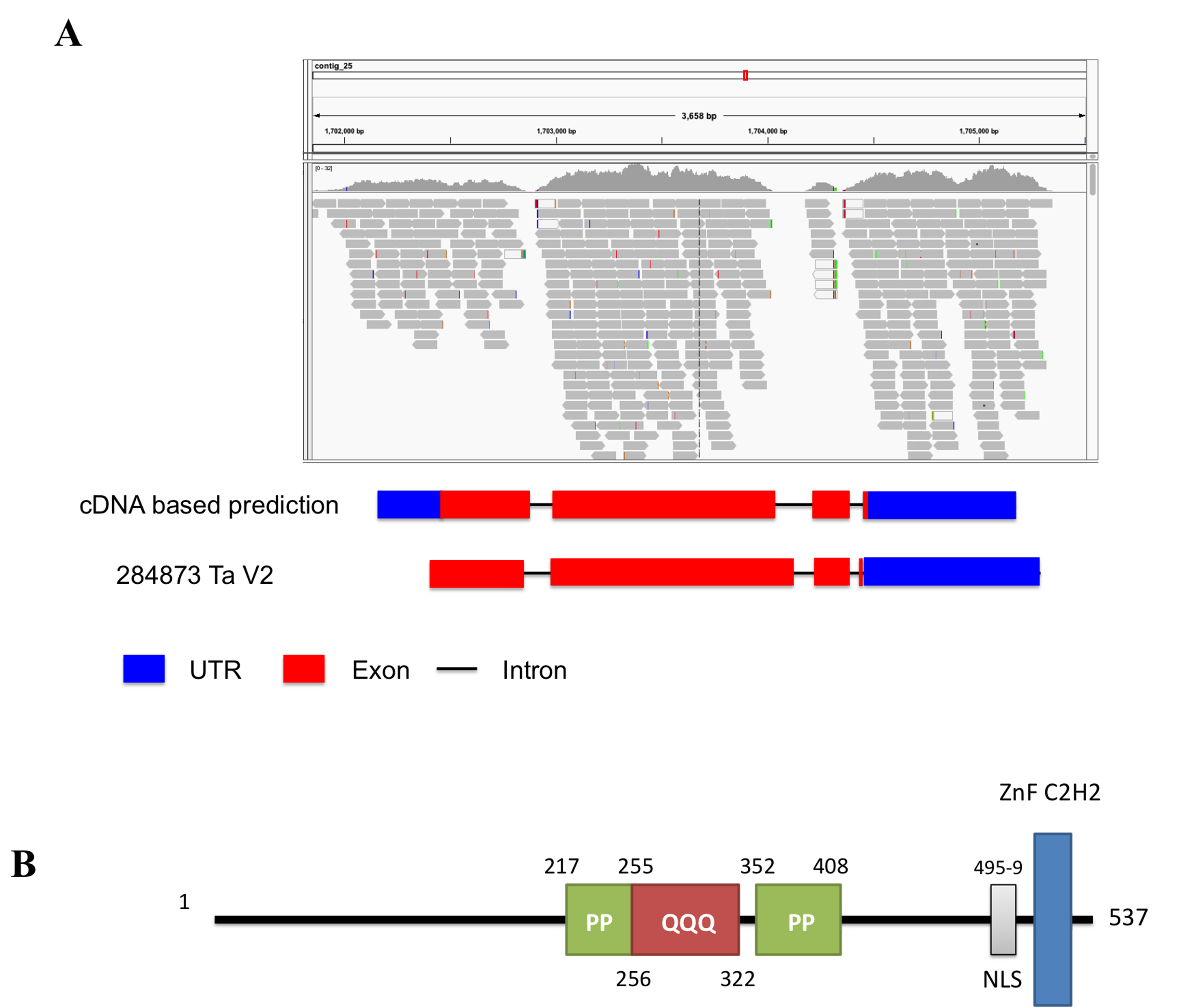

Supplement: Additional file 1: Figure S1. — A) Overview of 4500 bp of the blu7 gene locus showing the reads mapping to this region. The JGI Genwise predicted structure for the blu7 gene (ID 284873) and that derived from our cDNA clone are schematically represented. B) Schematic representation of the domains found for the Blu7 deduced protein. The scheme indicates the proline rich region (PP, green box), the putative activation domain (QQQ, red box), the nuclear localization signal (NLS, light grey box) and the C2H2 zinc finger domain (ZnF C2H2). Numbers indicate amino acid positions. The dotted box shows the sequence deleted as a result of the gene replacement event. (JPG 1968 kb) [file 12864_2016_2639_MOESM1_ESM.jpg]

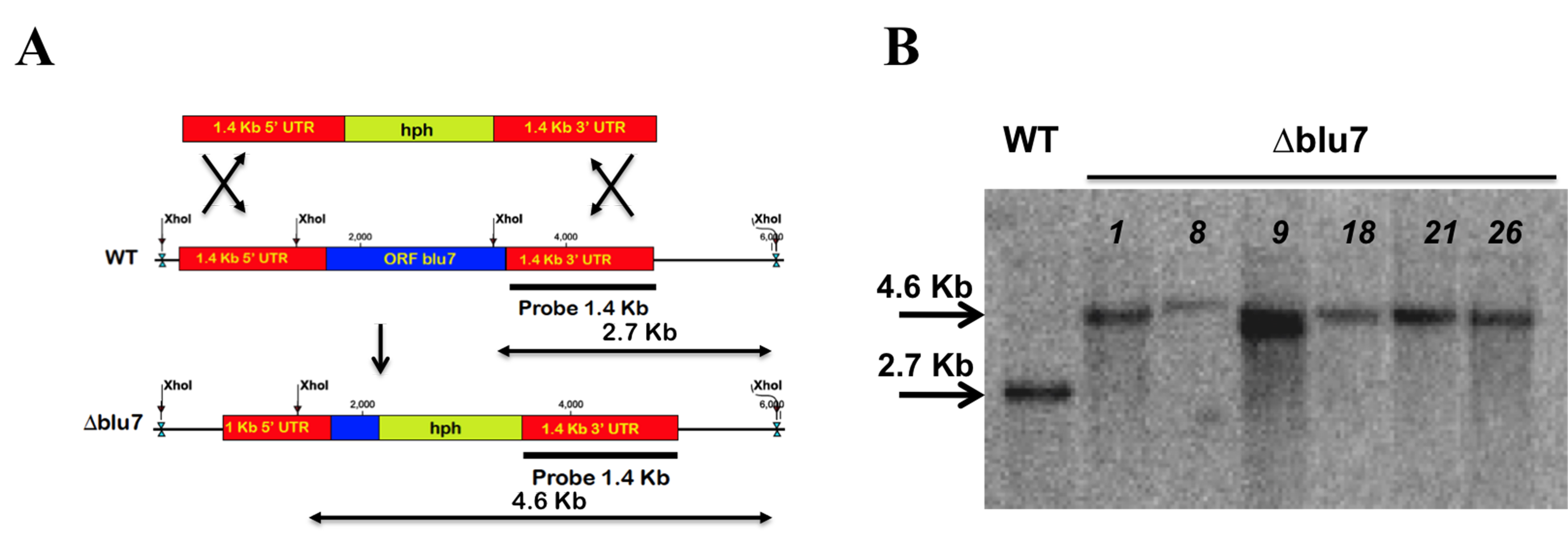

Supplement: Additional file 2: Figure S2. — Southern blot analysis of blu7 mutants. A) Schematic representation of the gene replacement event. Blue and red boxes represent the blu7 ORF and its 5’ & 3’ flanking regions, respectively; and restriction sites for XhoI, the enzyme used for DNA digestion indicated. The genes replacement construct is represented by the hygromycin resistance cassette (hph; green box), and the blu7 5’ & 3’ flanking regions (red boxes). The probe, a 1.4 kb-long segment corresponding to the 3’ UTR of the blu7 locus, is also indicated. B) Autoradiogram of the Southern analysis. The expected 2.7 kb signal for the WT and the 4.6 kb for the ∆blu7 is shown due to a loss of the XhoI site by the hph replacement. (JPG 873 kb) [file 12864_2016_2639_MOESM2_ESM.jpg]

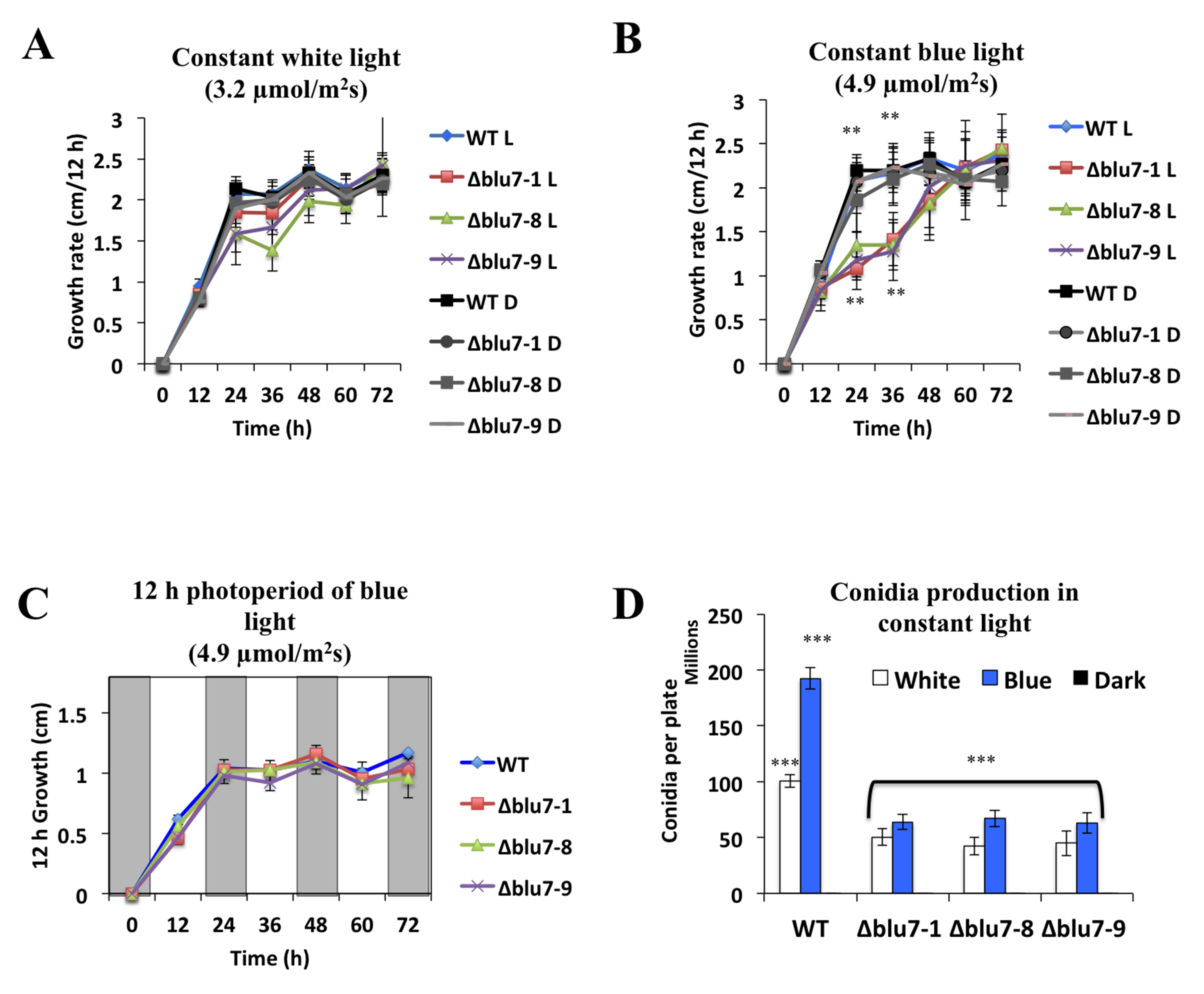

Supplement: Additional file 3: Figure S3. — Light growth response of the WT and ∆blu7 strains under constant illumination. A) Growth rate of WT and ∆blu7 mutant during 72 h in darkness (D) or constant white light. B) Growth rate of WT and ∆blu7 mutant during 72 h in constant blue light. C) Growth rate of the ∆blu7 mutant and WT under photoperiod of blue light- dark conditions during 72 h. D) Conidia production of the WT and ∆blu7 mutant under constant white (3.2 μmolm−2s−1) or blue (4.9 μmolm−2s−1) light treatments. One-way ANOVA and a pairwise t-test were applied to 6 independent replicates; asterisks indicate statistically significant differences (α < 0.05). (JPG 1758 kb) [file 12864_2016_2639_MOESM3_ESM.jpg]

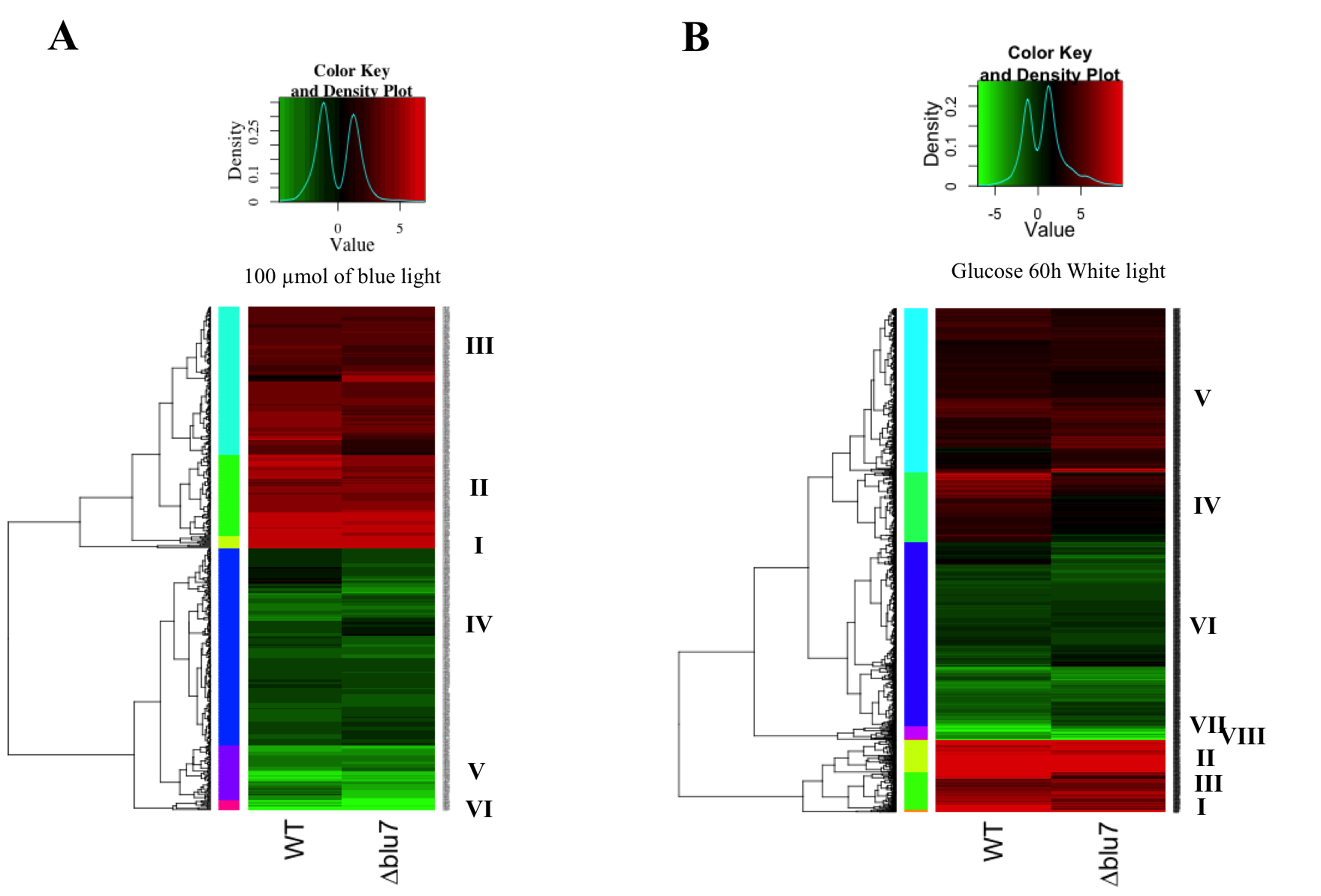

Supplement: Additional file 5: Figure S4. — Heatmap of the overall light regulated genes of WT and ∆blu7 mutant. Hierarchical clustering of the differential genes after a pulse of 100 μmolm−2 of blue light (A) or under constant white light (B) of the WT and ∆blu7 mutant is shown. (JPG 1801 kb) [file 12864_2016_2639_MOESM5_ESM.jpg]

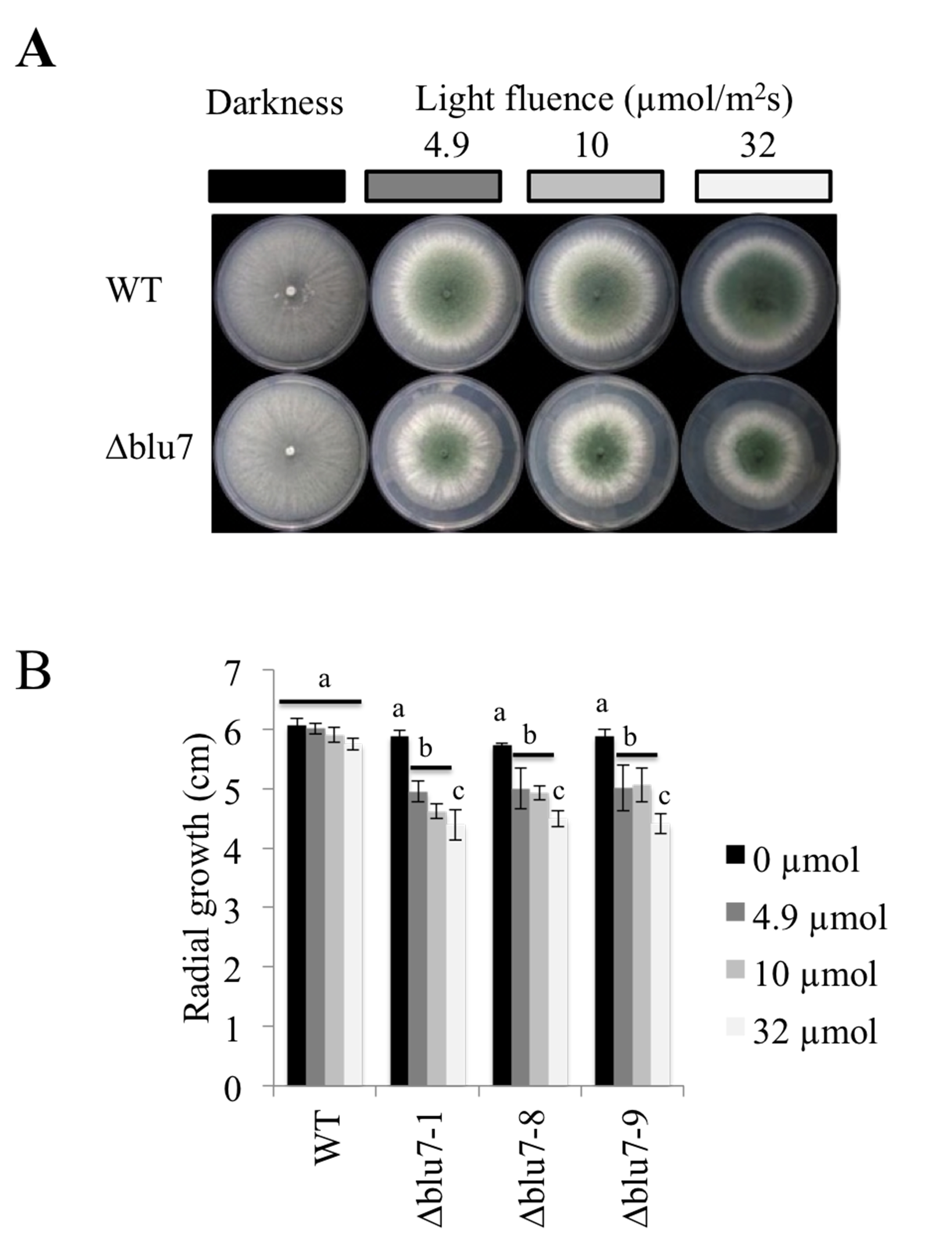

Supplement: Additional file 10: Figure S4. — A) Phenotype of the WT and ∆blu7 after under constant blue light of 4.9, 10 and 32 μmolm−2s−1. B) Radial colony growth after 72 h of light treatment of the WT and ∆blu7 strains. One-way ANOVA and a pairwise t-test were applied to data. Different letters indicate statistically significant differences (α < 0.05). (JPG 1062 kb) [file 12864_2016_2639_MOESM10_ESM.jpg]

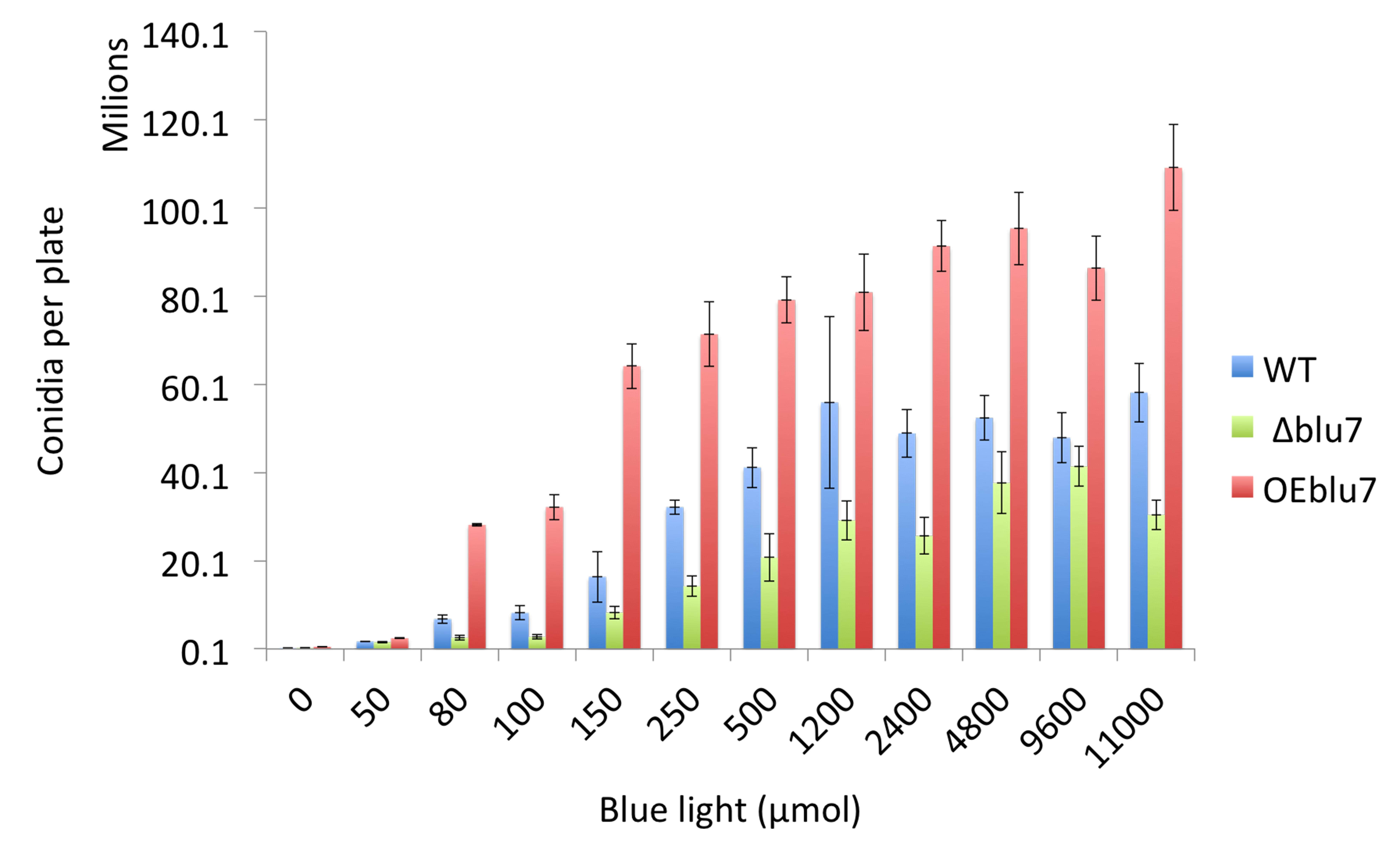

Supplement: Additional file 11: Figure S5. — Stimulation of photoconidiation by several blue light fluences (from 50 to 11000 μmolm−2) in the WT, ∆blu7 mutant and overexpressing (OEblu7) strains. (JPG 1487 kb) [file 12864_2016_2639_MOESM11_ESM.jpg]
